# Endogenous Protein Interactome of Human UDP-Glucuronosyltransferases Exposed by Untargeted Proteomics

**DOI:** 10.3389/fphar.2017.00023

**Published:** 2017-02-03

**Authors:** Michèle Rouleau, Yannick Audet-Delage, Sylvie Desjardins, Mélanie Rouleau, Camille Girard-Bock, Chantal Guillemette

**Affiliations:** Pharmacogenomics Laboratory, Canada Research Chair in Pharmacogenomics, Faculty of Pharmacy, Centre Hospitalier Universitaire de Québec Research Center, Laval UniversityQuébec, QC, Canada

**Keywords:** UGT, proteomics, protein-protein interaction, affinity purification, mass spectrometry, metabolism, human tissues

## Abstract

The conjugative metabolism mediated by UDP-glucuronosyltransferase enzymes (UGTs) significantly influences the bioavailability and biological responses of endogenous molecule substrates and xenobiotics including drugs. UGTs participate in the regulation of cellular homeostasis by limiting stress induced by toxic molecules, and by controlling hormonal signaling networks. Glucuronidation is highly regulated at genomic, transcriptional, post-transcriptional and post-translational levels. However, the UGT protein interaction network, which is likely to influence glucuronidation, has received little attention. We investigated the endogenous protein interactome of human UGT1A enzymes in main drug metabolizing non-malignant tissues where UGT expression is most prevalent, using an unbiased proteomics approach. Mass spectrometry analysis of affinity-purified UGT1A enzymes and associated protein complexes in liver, kidney and intestine tissues revealed an intricate interactome linking UGT1A enzymes to multiple metabolic pathways. Several proteins of pharmacological importance such as transferases (including UGT2 enzymes), transporters and dehydrogenases were identified, upholding a potential coordinated cellular response to small lipophilic molecules and drugs. Furthermore, a significant cluster of functionally related enzymes involved in fatty acid β-oxidation, as well as in the glycolysis and glycogenolysis pathways were enriched in UGT1A enzymes complexes. Several partnerships were confirmed by co-immunoprecipitations and co-localization by confocal microscopy. An enhanced accumulation of lipid droplets in a kidney cell model overexpressing the UGT1A9 enzyme supported the presence of a functional interplay. Our work provides unprecedented evidence for a functional interaction between glucuronidation and bioenergetic metabolism.

## Introduction

UDP-glucuronosyltransferases (UGTs) are well known for their crucial role in the regulation of cellular homeostasis, by limiting stress induced by toxic drugs, other xenobiotics and endogenous lipophilic molecules, and by controlling the hormonal signaling network (Rowland et al., 2013; Guillemette et al., 2014). UGTs coordinate the transfer of the sugar moiety of their co-substrate UDP-glucuronic acid (UDP-GlcA) to amino, hydroxyl and thiol groups on a variety of lipophilic molecules, thereby reducing their bioactivity and facilitating their excretion. In humans, nine UGT1A and ten UGT2 enzymes constitute the main glucuronidating enzymes. UGTs are found in nearly all tissues, each UGT displaying a distinct profile of tissue expression, and are most abundant in the liver, kidney and gastrointestinal tract, where drug metabolism is highly active. These membrane-bound enzymes localized in the endoplasmic reticulum (ER) share between 55 and 97% sequence identity, thus displaying substrate specificity and some overlapping substrate preferences (Rowland et al., 2013; Guillemette et al., 2014; Tourancheau et al., 2016). For instance, the alternative first exons of the single *UGT1* gene produce the nine UGT1A enzymes with distinct N-terminal substrate binding domains but common C-terminal UDP-GlcA-binding and transmembrane domains. The seven UGT2B enzymes and UGT2A3 are encoded by eight distinct genes, whereas UGT2A1 and UGT2A2 originate from a single gene by a UGT1A-like, alternative exon 1 strategy. However, similar to UGT1As, substrate binding domains of UGT2 enzymes are more divergent than their C-terminal domains.

Genetic variations, epigenetic regulation, as well as post-transcriptional and translational modifications, all contribute to the modulation of UGT conjugation activity, thereby influencing an individual's response to pharmacologic molecules and the bioactivity of endogenous molecules (Guillemette et al., 2010, 2014; Ramírez et al., 2010; Hu et al., 2014; Dluzen and Lazarus, 2015). For instance, genetic lesions at the *UGT1* locus that impair UGT1A1 expression or activity result in transient or fatal hyperbilirubinemia, characterizing Gilbert and Crigler-Najjar syndromes, respectively (Costa, 2006).

Several lines of evidence support protein-protein interactions (PPIs) among UGTs and with other enzymes of pharmacological importance (Taura et al., 2000; Fremont et al., 2005; Takeda et al., 2005a,b, 2009; Ishii et al., 2007, 2014; Operaña and Tukey, 2007). These interactions may also significantly influence UGT enzymatic activity (Bellemare et al., 2010b; Ménard et al., 2013; Ishii et al., 2014; Fujiwara et al., 2016). In addition, interactions of UGT proteins with some anti-oxidant enzymes that have been recently uncovered have raised the interesting concept of alternative functions of UGTs in cells (Rouleau et al., 2014). However, most studies have been conducted in cell-based systems with overexpression of tagged UGTs and little evidence in human tissues supports the extent of this mechanism and its physiological significance.

PPIs are essential to cell functions including responses to extracellular and intracellular stimuli, protein subcellular distribution, enzymatic activity, and stability. Understanding molecular interaction networks in specific biological contexts is therefore highly informative of protein functions. We aimed to gain insight on the endogenous protein interaction network of UGT1A enzymes by applying an unbiased proteomics approach in main drug metabolizing human tissues. In doing so, we provide support to a potential coordinated cellular response to small lipophilic molecules and drugs. Importantly, a potential functional interplay between UGT1A enzymes and those of bioenergetic pathways also emerges from this exhaustive endogenous interaction network.

## Materials and methods

### UGT1A enzyme antibodies

The anti-UGT1A rabbit polyclonal antibody (#9348) that specifically recognizes UGT1A enzymes, and not the alternative UGT1A variant isoforms 2, has been described (Bellemare et al., 2011). Purification was performed using the biotinylated immunogenic peptide (K_520_KGRVKKAHKSKTH_533_; Genscript, Piscataway, NJ, USA) and streptavidin magnetic beads (Genscript) per the manufacturer's instructions. Antibodies (3 ml) were incubated O/N at 4°C with peptide-streptavidin beads, and then washed with PBS to remove unbound immunoglobulins. UGT1A-specific antibodies were eluted using glycine (0.125 M, pH 2.9), and rapidly buffered with Tris pH 8.0. Purified antibodies were subsequently concentrated using a centrifugal filter unit (cut off 3 kDa; Millipore (Fisher Scientific), Ottawa, ON) to a final volume of 1 ml.

### Affinity purification of endogenous UGT1A enzymes and their interacting partners in human tissues and a UGT1A expressing cellular model

Human liver, kidney and intestine S9 fractions comprised of ER and associated membranes as well as cytosolic cellular content (Xenotech LLC, Lenexa, KS, USA) were from 50, 4, and 13 donors, respectively. This study was reviewed by the local ethics committee and was exempt given that anonymized human tissues were from a commercial source. Human colon cancer HT-29 cells (ATCC, Manassas, VA, USA) were grown in DMEM supplemented with 10% fetal bovine serum (Wisent, St-Bruno, QC, Canada), 50 mg/ml streptomycin, 100 IU/ml penicillin, at 37°C in a humidified incubator with 5% CO_2_ as recommended by ATCC. Immunoprecipitations (IP) were conducted according to standard procedures (Savas et al., 2011; Ruan et al., 2012), with at least three independent replicates per sample source. For each sample, 1 mg protein was lysed in 1 ml lysis buffer A [final concentration: 50 mM Tris-HCl pH 7.4, 150 mM NaCl, 0.3% deoxycholic acid, 1% Igepal CA-630 (Sigma-Aldrich), 1 mM EDTA, complete protease inhibitor (Roche, Laval, QC, Canada)] for 45 min on ice. This buffer included deoxycholate to enhance membrane solubilization and stringency of immunoprecipitation conditions. Lysates were then homogenized by pipetting up and down through fine needles (18G followed by 20G) 10–20 times on ice. Lysates were cleared of debris by centrifugation for 15 min at 13,000 g. UGT1A enzymes were immunoprecipitated from cleared lysates with 4 μg of purified anti-UGT1A for 1 h at 4°C with end-over-end agitation. After addition of protein G–coated magnetic beads (200 μl Dynabeads, Life Technologies, Burlington, ON), lysates were incubated O/N at 4°C. Beads were washed three times with 1 ml lysis buffer A and subsequently processed for mass spectrometry (MS) analysis, as described below. Control IPs were conducted in similar conditions using 4 μg normal rabbit IgGs (Sigma-Aldrich) per protein sample. The inclusion of 150 mM NaCl and 0.3% deoxycholate ensured stringent wash conditions.

### Liquid chromatography-MS/MS identification of UGT1A interacting partners

Protein complexes bound to magnetic beads were washed 5 times with 20 mM ammonium bicarbonate (1 ml). Tryptic digestion and desalting was performed as described (Rouleau et al., 2016). Briefly, bead-bound proteins were digested in 10 μg/μl trypsin for 5 hrs at 37°C. The tryptic digest was recovered, dried, and resuspended in 30 μl sample buffer (3% acetonitrile, 0.1% trifluoroacetic acid, 0.5% acetic acid). Peptides were desalted on a C18 Empore filter (ThermoFisher Scientific), dried out, resuspended in 10 μl 0.1% formic acid and analyzed using high-performance liquid chromatography-coupled MS/MS on a LTQ linear ion trap-mass spectrometer equipped with a nanoelectrospray ion source (Thermo Electron, San Jose, CA, USA) or on a triple-quadrupole time-of-flight mass spectrometer (TripleTOF 5600, AB Sciex, Concord, ON) as described (Rouleau et al., 2014). Data files were submitted for simultaneous searches using Protein Pilot version 4 software (AB Sciex) utilizing the Paragon and Progroup algorithms (Shilov). The RAW or MGF file created by Protein Pilot was used to search with Mascot (Matrix Science, London, UK; version 2.4.1). Mascot was set up to search against the human protein database (Uniref May 2012; 204083 entries) supplemented with a complete human UGT protein sequence database comprised of common UGT coding variations and protein sequences of newly discovered alternatively spliced UGT isoforms (assembled in-house November 2013; 882 entries). Mascot analysis was conducted using the following settings: tryptic peptides, fragment and parent ion tolerance of 0.100 Da, deamidation of asparagine and glutamine and oxidation of methionine specified as variable modifications, deisotoping was not performed, two missed cleavage were allowed. Mass spectra were also searched in a reversed database (decoy) to evaluate the false discovery rate (FDR). On-beads digestion and MS analyses were performed by the proteomics platform of the CHU de Québec Research Center. The MS proteomics data have been deposited to the ProteomeXchange Consortium (http://proteomecentral.proteomexchange.org) via the PRIDE partner repository with the dataset identifier PXD000295.

Identification of proteins in Scaffold (version 4.6.1; Proteome Software, Portland, OR) was carried out using two sets of criteria: (1) for UGT proteins, 95% peptide and protein probability, and 1 unique peptide were used, considering the high level of sequence identity among the proteins in this family. For the same reason of high sequence identity, each identified peptide was manually assigned to the proper UGT protein or to the common UGT sequence (Supplementary Table 1). (2) For UGT1A interacting proteins, specificity threshold was set to 95% peptide and protein probability and a minimum of 2 unique peptides. Proteins that contained similar peptides and could not be differentiated based on MS/MS analysis alone were grouped to satisfy the principles of parsimony. Detailed proteomics datasets are provided in Supplementary Tables 4–7.

Confidence scores of each UGT1A-protein interaction were determined using the computational tools provided online at http://crapome.org (Choi et al., 2011). Spectral counts for each identified protein were normalized to the length of the protein and total number of spectra in the experiment. Two empirical scores (FC-A and more stringent FC-B) and one probability score (SAINT) (Mellacheruvu et al., 2013) were then calculated based on normalized spectral counts of identified proteins in UGT1A immunoprecipitation samples compared to our matching control immunoprecipitation samples (CRAPome Workflow 3). Confidence score calculations were conducted separately for each tissue, with the following *analysis options*: FC-A: Default parameters; FC-B: *User* controls, *stringent* background estimation, *geometric* combining replicates; SAINT: *User* Controls, *Average - best 2* Combining replicates, *10* Virtual controls and *default* SAINT options. Confidence scores for all UGT1A interaction partners are given in Supplementary Table 2.

### Bioinformatics tools and data analysis

The common external contaminants keratins and trypsin were manually removed from the lists of interacting proteins prior to pathway enrichment analysis. UGT1A interacting partners were classified according to KEGG pathways (update November 12, 2016) using ClueGO and CluePedia Apps (v2.3.2) in Cytoscape 3.4 (Bindea et al., 2009, 2013). Enrichment was determined based on a two-sided hypergeometric statistical test and a Bonferroni step down correction method. Only enriched pathways with *P* < 0.05 and a Kappa score threshold of 0.4 were considered. The following optional criteria were also used for the search: minimum # genes = 4, minimum 2% genes. The UGT1A interactome was generated using Cytoscape basic tools. Because protein annotations based on tools such as KEGG and Gene Ontology are partial, the UGT1A interactome was subsequently manually extended to include significant UGT1A interactors that were absent in the original output but involved in enriched pathways, per their Uniprot entries (www.uniprot.org) and literature mining. Details are included in the legend of **Figure 3**.

### Validation of protein-protein interactions by Co-IP and immunofluorescence (IF)

HEK293 cells stably expressing the human enzyme UGT1A9-myc/his (a pool of cells) were used (Bellemare et al., 2010a). Expression and glucuronidation activity of the tagged UGT1A9 in this model have been described and were similar to the untagged enzyme (Bellemare et al., 2010a). In the current study, only the myc tag served for UGT1A9 detection and the his tag was not exploited. Cells were transfected with Lipofectamine 2000 (Life Technologies) to transiently express tagged protein partners. HA-ACOT8 and FLAG-SH3KBP1 were kindly provided by Dr Ming-Derg Lai (National Cheng Kung University, Taiwan; Hung et al., 2014) and Dr Mark McNiven (Mayo Clinic, Rochester, MN; Schroeder et al., 2010) respectively. The PHKA2-myc-FLAG expression construct was purchased from OriGene (Rockville, MD, USA).

Co-IP: HEK293 cells (3 × 10^5^ cells plated in 10 cm dishes) were harvested 40 h post-transfection. Cells were washed three times with PBS, lysed in 800 μl lysis buffer B (50 mM Tris-HCl pH 7.4, 150 mM NaCl, 1% Igepal, 1 mM DTT, complete protease inhibitor) for 1 h at 4°C and subsequently homogenized and centrifuged as described above. Immunoprecipitation with purified anti-UGT1A antibodies (2 μg) or control rabbit IgG (2 μg) and 50 μl Protein-G magnetic beads was as above. Protein complexes were washed three times in lysis buffer B and eluted in Laemmli sample buffer by heating at 95°C for 5 min. Eluates were subjected to SDS-PAGE, and the presence of interacting partners was revealed by immunoblotting using anti-tag antibodies specified in figures and legends: anti-myc (clone 4A6, EMD Millipore, Etobicoke, ON, Canada; 1:5000), anti-FLAG (clone M2, Sigma-Aldrich, St-Louis, MO, USA; 1:20 000) and anti-HA (Y-11, Santa Cruz Biotechnologies, Dallas, TX, USA; 1:500).

IF: HEK293 cells (2 × 10^5^ cells per well of 6-well plates) grown on coverslips were harvested 36 h post-transfection and processed for IF, as described (Rouleau et al., 2016). ACOT8 was detected with anti-HA (1:500), SH3KBP1 with anti-FLAG (1:1500), UGT1A9-myc/his with anti-myc (1:200) or purified anti-UGT1A (1:500), and with secondary goat anti-rabbit, goat anti-mouse or donkey anti-mouse respectively, conjugated to either AlexaFluor 488 or 594 (1:1000; Invitrogen). Immunofluorescence images were acquired on a LSM510 META NLO laser scanning confocal microscope (Zeiss, Toronto, ON, Canada). Zen 2009 software version 5.5 SP1 (Zeiss) was used for image acquisitions.

### Quantification of lipid droplets

HEK293 cells grown on coverslips were fixed in 3.7% formaldehyde (Sigma) for 30 min at RT. Cells were then gently washed three times with PBS and incubated for 10 min in 0.4 μg/mL Nile Red (Sigma). After being rinsed three times, coverslips were mounted on glass slides using Fluoromount (Sigma) as a mounting medium. Images were acquired on a Wave FX-Borealis (Quorum Technologies, Guelph, ON, Canada) - Leica DMI 6000B (Clemex Technologies inc., Longueil, QC, Canada) confocal microscope, with a 491 nm laser and 536 nm filter. Z-stacks were acquired every 0.15 μm. Stacks were analyzed using ImageJ (v1.51f; U.S. National Institutes of Health, Bethesda, MD, USA) and the 3-D Object Counter plugin (Bolte and Cordelieres, 2006). Results are derived from 3 independent experiments and more than 140 cells per experiment were analyzed for each condition. Fluorescence images were acquired on an LSM 510 microscope as above.

## Results

### Endogenous UGT1A enzymes associate with several other metabolic proteins in non-malignant human tissues

**Figure 1:**
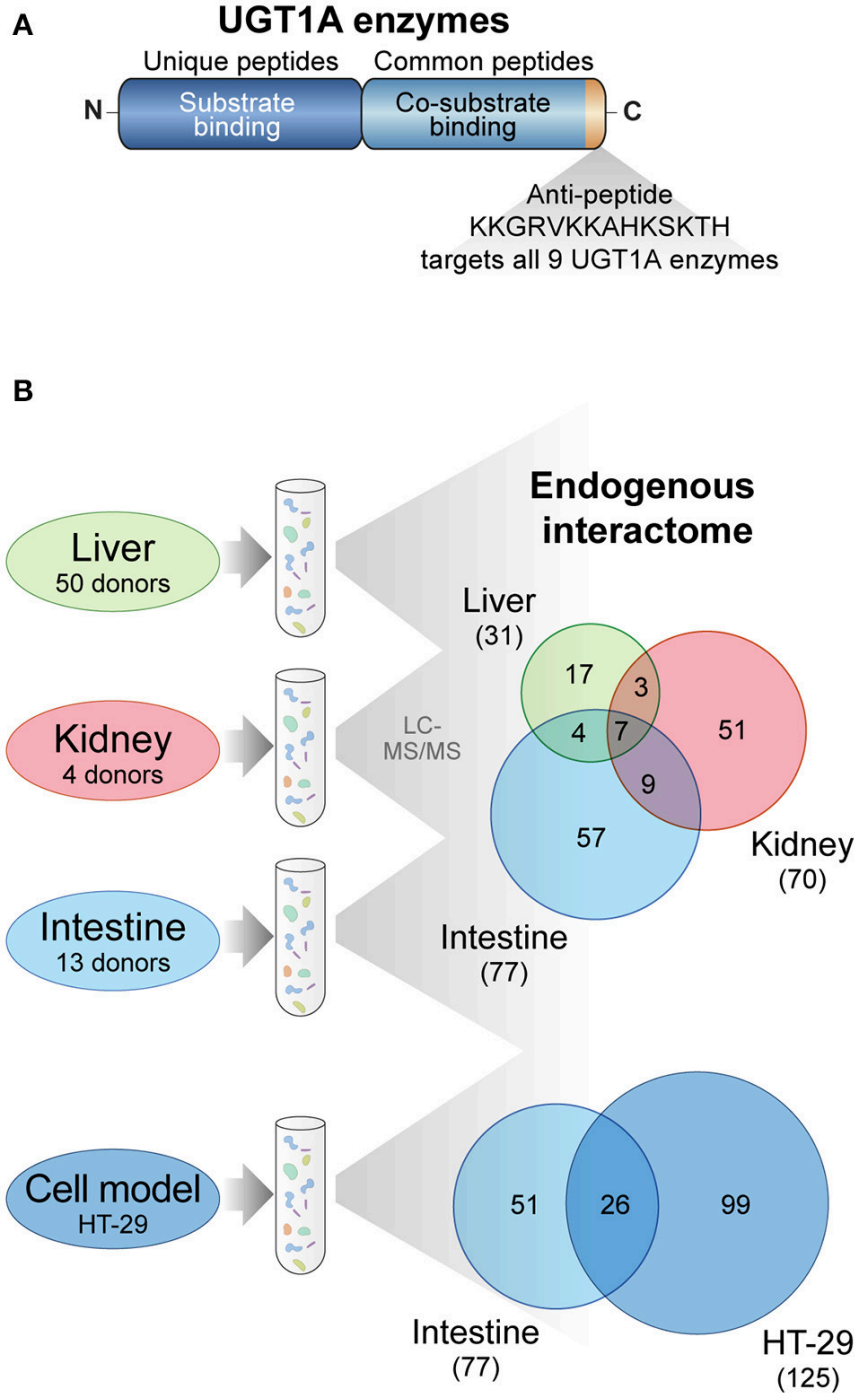
**UGT1A interaction network investigated by untargeted proteomics. (A)** The nine UGT1A enzymes are distinguished by the amino acid sequence of their substrate binding domain (unique peptides) whereas they share identical C-terminal co-substrate and transmembrane domains (common peptides). The anti-UGT1A antibody used in this study was raised against a C-terminal peptide common to all nine UGT1A enzymes but does not recognize the main spliced alternative isoforms 2 or UGT1A_i2s. **(B)** Experimental approach to establish endogenous UGT1A protein interactomes in drug metabolizing tissues and in the colon cancer cell model HT-29. Immunoprecipitation of UGT1A enzymes was conducted with the anti-UGT1A antibody. The numbers of common and unique UGT1A protein partners identified by mass spectrometry and above confidence threshold are represented in the Venn diagrams. Datasets were established on a minimum of two biological replicates. A Venn diagram for the 4 matrices is presented in Supplementary Figure 2. A list of proteins in each group is provided in Supplementary Table 3.

The endogenous interactome of human UGT1A enzymes was established in three major metabolic tissues, namely liver, kidney and intestine from pools of 4–50 donors, using S9 tissue fractions comprised of ER and associated membranes as well as cytosolic cellular content (Figure 1). IPs were conducted with an antibody specific to the C-terminal region common to the nine human UGT1A enzymes, thereby allowing affinity purification of all UGT1A enzymes expressed in studied tissues (Figure 1A). This antibody was shown by western blotting to lack affinity for alternatively spliced UGT1A isoform 2 proteins derived from the same human *UGT1* gene locus (Bellemare et al., 2011). The experimental approach to establish the endogenous UGT1A enzymes interactome using the anti-UGT1A enzymes antibody is presented in Figure 1B.

**Figure 2:**
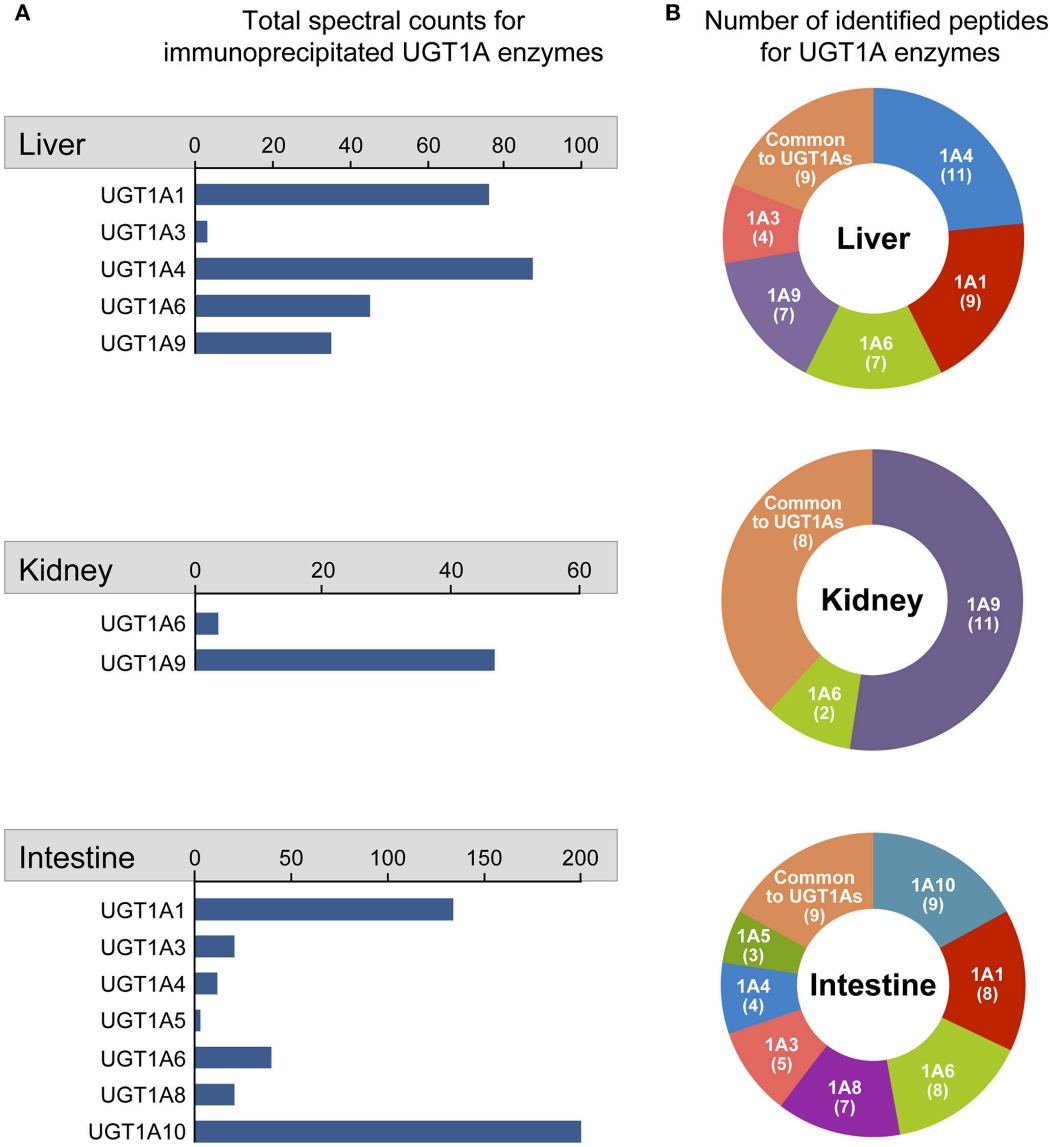
**Quantitative overview of UGT1A enzymes immunoprecipitated from each tissue**. Identification of immunoprecipitated UGT1A enzymes was based on the detection of peptides unique to specified UGT1A enzymes. **(A)** The quantitative assessment of each immunoprecipitated UGT1A is given by the total number of spectral counts for peptides unique to each UGT1A identified by mass spectrometry. Total spectral counts for peptides common to all UGT1A enzymes (Liver: 465; Kidney: 67; Intestine: 1561) were not considered in the quantitative assessment of specific UGT1As. **(B)** For each tissue, the number of peptides unique to each UGT1A identified by MS/MS analysis is represented in ring charts. Detailed quantification and unique/common UGT1A peptides identified are presented in Supplementary Table 1.

Multiple UGT1A enzymes were immunopurified from each tissue in line with their documented expression profile (Figure 2). The list of specific UGT1A enzymes immunoprecipitated from each tissue was established based on their unique N-terminal peptide sequences, whereas multiple additional peptides corresponding to the common C-terminal half of the UGT1A proteins and thereby common to all UGT1A enzymes were also observed (Figure 2B, Supplementary Figure 1; Supplementary Table 1).

Spectral counts for unique peptides provided a quantitative appreciation of immunoprecipitated UGT1A (Figure 2A). UGT1A1 and UGT1A4 were the most abundant UGT1As in hepatic IPs, whereas UGT1A1 and UGT1A10 were predominantly immunopurified from the intestine and UGT1A9 from the kidney (Figure 2A, Supplementary Table 1). UGT1A9 (*n* = 51 spectra) was far more abundant than UGT1A6 (*n* = 2 spectra) in the kidney whereas UGT1A10 (*n* = 194 spectra) predominated over most other UGT1A in the intestine, although all UGT1A enzymes were identified besides UGT1A7 and UGT1A9. These metrics indicated that an exhaustive immunoprecipitation of UGT1As from each tissue was achieved.

### UGT1A interaction network and functional annotation

A total of 9 independent AP-MS datasets (4 liver, 3 kidney, and 2 intestine replicates of control and UGT1A AP-MS) efficiently immunoprecipitated UGT1A enzymes and associated proteins. Mass spectra were assigned to specific proteins using Mascot and Scaffold software. A list of UGT1A-interacting proteins was created based on the analysis of total spectral counts assigned to each identified protein in each replicate to obtain empirical (FC-B) and probability (SAINT) confidence scores (Supplementary Table 2). Using a FC-B score threshold of 1.42, we reported a total of 148 proteins forming endogenous interactions with UGT1A enzymes in the three surveyed human tissues (31 in the liver, 70 in the kidney and 77 in the intestine) (Figure 1B, Supplementary Table 2). This FC-B threshold was selected based on the validated protein partner having the lowest probability score, corresponding to PHKA2 in the intestine (see below). This approach was chosen because of the inherent difficulty to obtain similar replicate datasets with AP-MS from tissues, especially in intestine, a variability highly penalized in the SAINT scoring algorithm (Supplementary Figure 2). To further strengthen the UGT1A interactome in the gastrointestinal tract, we also conducted three more replicate AP-MS experiments of endogenous UGT1A enzymes with the human colon cancer cell line HT-29, expressing high levels of UGT1As. The intestinal UGT expression profile is well represented in HT-29 cells, with UGT1A1, UGT1A6, UGT1A8, and UGT1A10 immunoprecipitated in similar proportions (Supplementary Table 1). Using the FC-B threshold used for tissues (1.42), 125 interaction partners were selected for further analysis. Of those, 44 proteins were common with those immunoprecipitated in non-malignant tissue samples, including 26 common with the intestine dataset (Figure 1B, Supplementary Figure 3). UGT1A protein partners with highest significance scores are given in Table 1 whereas a complete list of immunoprecipitated protein partners is provided in Supplementary Table 2.

**Table 1:** **Top 10 UGT1A interaction partners^a^ for each tissue based on confidence score**.

**Liver**				**Kidney**				**Intestine**			
**Protein name^b^**	**Coverage (%)^c^**	**Total spectral counts^d^**	**FC_B score**	**Protein name^b^**	**Coverage (%)^c^**	**Total spectral counts^d^**	**FC_B score**	**Protein name^b^**	**Coverage (%)^c^**	**Total spectral counts^d^**	**FC_B score**
**PHKB**	36	193	28.55	TOP2B	16	53	5.93	ATP5A1	13	8	4.91
**PHKA2**	34	180	26.62	PFKL	24	35	4.68	UGT2A3	24	61	4.63
PHKG2	40	68	10.49	TRA2B	24	36	4.21	**GBF1**	12	60	4.60
**PRDX2**	47	55	7.62	ATP5A1	33	32	4.10	**SLC25A5**	35	54	4.37
**UGT2B7**	19	31	5.53	**PRDX2**	41	50	3.60	RALGAPB	13	51	4.26
**PRDX1**	35	25	3.42	**PRDX1**	44	39	3.08	**PRDX2**	24	46	4.06
**ECH1**	28	17	2.61	HSPA8	30	21	2.70	**PRDX1**	31	45	4.01
**SLC25A5**	17	9	2.20	SLC34A2	16	17	2.66	RALGAPA2	6	29	3.28
**GBF1**	4	9	2.15	ACCA2	43	21	2.30	**ECH1**	30	21	2.84
UGT2B4	11	12	1.98	ASS1	42	21	2.22	PDIA3	5	3	2.82

a *Excluding common IP protein contaminants (structural, ribosomal and RNA-binding proteins)*.

b *Proteins in bold were identified in the 3 tissues*.

c *Total coverage calculated with peptides identified in all replicates (n = 4, 3 and 2 for the liver, kidney and intestine, respectively)*.

d *Total spectral counts of all replicates*.

To portray the global functions enriched in the UGT1A interactome, the UGT1A protein partners from the three surveyed tissues were classified per the KEGG pathway database. Structural proteins such as tubulins, myosins, actin, as well as multiple ribosomal protein subunits (RPL/RPS proteins) and other RNA-binding proteins involved in mRNA splicing (e.g., heterogeneous ribonucleotide proteins (hnRNPs) and serine/arginine-rich splicing factors (SRSF) proteins) were significant classes of proteins immunoprecipitated with UGT1As. However, because these proteins are frequently non-specifically enriched in AP-MS experiments (Mellacheruvu et al., 2013), the specificity of interactions with UGT1A will require validation and will not be discussed further.

**Figure 3:**
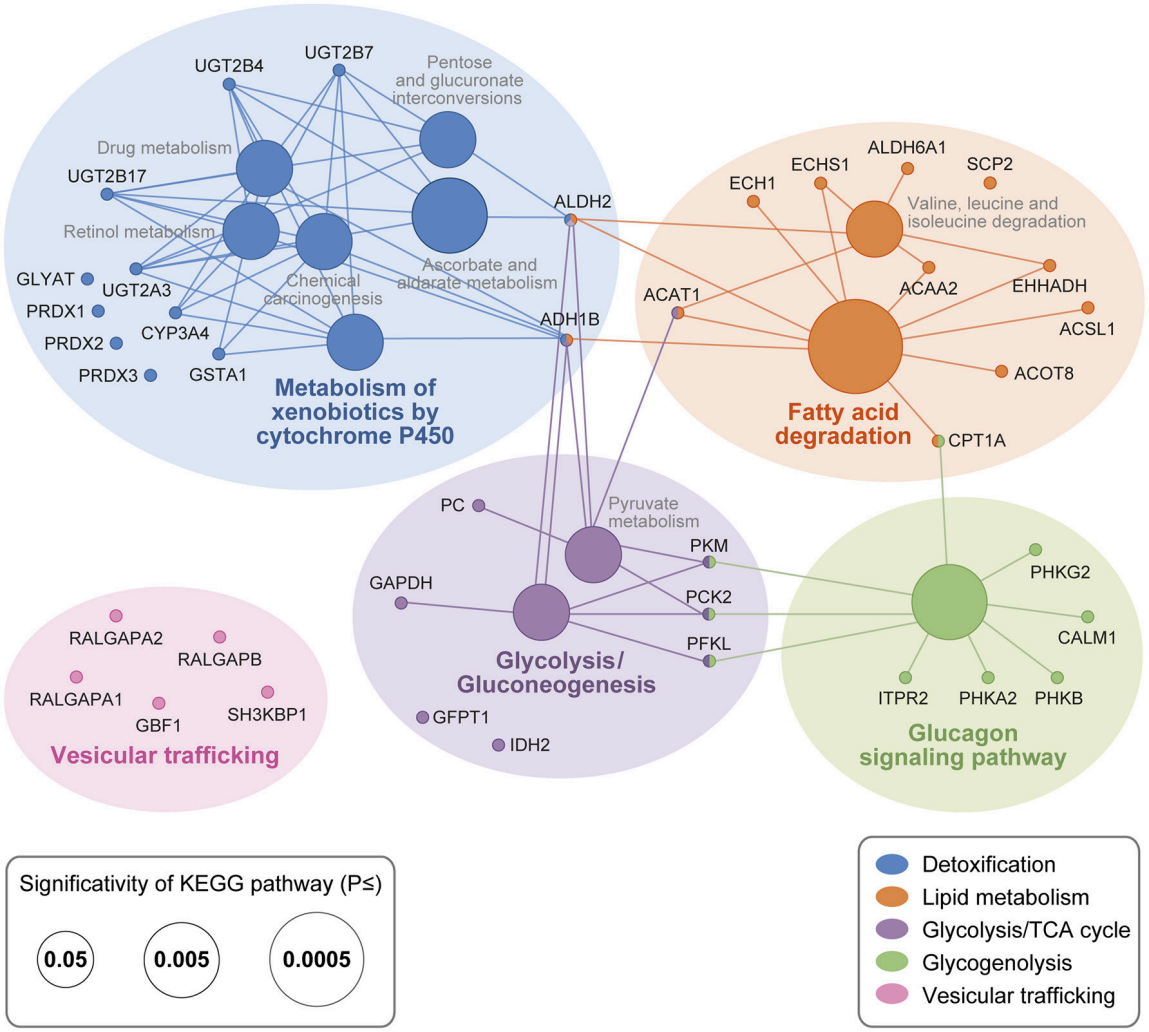
**UGT1A interaction network in drug metabolizing tissues**. UGT1A interacting proteins were classified according to KEGG pathways with ClueGO/CluePedia (Bindea et al., 2009, 2013). Node size is representative of pathway enrichment significance. Interactome was enhanced with significant interaction partners not part of KEGG pathways that are functionally related based on Uniprot and literature. These proteins are not linked to nodes but are grouped according to global functions. Structural proteins, ribosomal protein subunits and other RNA-binding proteins involved in mRNA splicing are not shown but were significantly enriched in UGT1A IPs. Full protein names are provided in Table 2. Complete lists of UGT1A interacting proteins are provided in Supplementary Table 2.

The interactome of UGT1A enzymes is characterized by numerous metabolic proteins playing roles in detoxification and bioenergetic pathways (Figure 3). They include the UGT2 glucuronosyltransferases UGT2A3, UGT2B4, UGT2B7, and UGT2B17, the glutathione S-transferase GSTA1, glycine N-acyltransferase GLYAT, the alcohol dehydrogenase ALDH2 and the antioxidant enzymes PRDX1 and PRDX2 (full protein names are provided in Table 2). Given their functions in line with high scoring proteins, ADH1B and PRDX3 were also included in the final interactome, having confidence interaction scores just below threshold (FC-B = 1.37; Supplementary Figure 2). Similarly, the cytochrome P450 CYP3A4 was included because also observed in liver tissue with a single high confidence peptide and a previously observed interaction partner (Fremont et al., 2005; Ishii et al., 2014). Enzymes of the lipid metabolism pathway were also significantly represented and most particularly several peroxisomal and mitochondrial proteins involved in fatty acid β-oxidation, namely ACOT8, ECH1, CPT1A, and ACAA2. To encompass all potential protein partners involved in lipid metabolism, a pathway that was functionally validated at a later stage (see below), relevant but slightly lower scoring proteins were incorporated in the final interactome, namely SCP2, ACSL1, EHHADH, ACAT1, and ECHS1 (FC-B = 1.38–1.19; Supplementary Figure 2). Finally, the glycolysis/pyruvate and glycogenolysis metabolic pathways were also significantly enriched, given the high number of immunoprecipitated UGT1A partners in these pathways. Several other protein partners, including transporters (SLC25A5, SLC25A13, and SLC34A2) and proteins participating in vesicular trafficking (RALGAPA1, RALGAPA2, RALGAPB, and GBF1) were also immunoprecipitated from tissues and may represent important partners (Table 1, Figure 3). The interaction network of UGT1A enzymes established in the HT-29 cell model was consistent with that built from tissues, with enrichments in xenobiotic and bioenergetics metabolic pathways. Several transporters, anti-oxidant, lipid metabolism, glycolytic/glycogen metabolic enzymes and vesicular trafficking proteins were all significantly identified in AP-MS on cells, as in tissues (Supplementary Table 2), further supporting the significance of the endogenous interactome of UGT1A enzymes.

**Table 2:** **Complete names of most significant UGT1A protein partners^a^**.

**Protein Names^b^**	
**Abbreviation**	**Complete names**
ACAA2	3-ketoacyl-CoA thiolase, mitochondrial
ACAT1/SOAT1	Sterol O-acyltransferase 1
ACOT8	Acyl-coenzyme A thioesterase 8
ACSL1	Long-chain-fatty-acid–CoA ligase 1
ADH1B	Alcohol dehydrogenase 1B
ALDH2	Aldehyde dehydrogenase, mitochondrial
ALDH6A1	Methylmalonate-semialdehyde dehydrogenase [acylating], mitochondrial
ASS1	Argininosuccinate synthase
ATP5A1	ATP synthase subunit alpha, mitochondrial
CALM1	Calmodulin
CPT1A	Carnitine O-palmitoyltransferase 1, liver isoform
CYP3A4	Cytochrome P450 3A4
ECH1	Delta(3,5)-Delta(2,4)-dienoyl-CoA isomerase, mitochondrial
ECHS1	Enoyl-CoA hydratase, mitochondrial
EHHADH	Peroxisomal bifunctional enzyme
GAPDH	Glyceraldehyde-3-phosphate dehydrogenase
GBF1	Golgi-specific brefeldin A-resistance guanine nucleotide exchange factor 1
GLYAT	Glycine N-acyltransferase
GSTA1	Glutathione S-transferase A1
HSPA8	Heat shock cognate 71 kDa protein
IDH2	Isocitrate dehydrogenase [NADP], mitochondrial
ITPR2	Inositol 1,4,5-trisphosphate receptor type 2
PC	Pyruvate carboxylase, mitochondrial
PCK2	Phosphoenolpyruvate carboxykinase [GTP], mitochondrial
PDIA3	Protein disulfide-isomerase A3
PFKL	ATP-dependent 6-phosphofructokinase, liver type
PHKA2	Phosphorylase b kinase regulatory subunit alpha, liver isoform
PHKB	Phosphorylase b kinase regulatory subunit beta
PHKG2	Phosphorylase b kinase gamma catalytic chain, liver/testis isoform
PKM	Pyruvate kinase
PRDX1	Peroxiredoxin-1
PRDX2	Peroxiredoxin-2
PRDX3	Peroxiredoxin-3
RALGAPA1	Ral GTPase-activating protein subunit alpha-1
RALGAPA2	Ral GTPase-activating protein subunit alpha-2
RALGAPB	Ral GTPase-activating protein subunit beta
SCP2	Non-specific lipid-transfer protein
SH3KBP1	SH3 domain-containing kinase-binding protein 1
SLC25A13	Calcium-binding mitochondrial carrier protein Aralar2
SLC25A5	ADP/ATP translocase 2
SLC34A2	Sodium-dependent phosphate transport protein 2B
TOP2B	DNA topoisomerase 2-beta
TRA2B	Transformer-2 protein homolog beta

a *Complete list of immunoprecipitated proteins is provided in Supplementary Table 2*.

b *Protein names are according to Uniprot (www.uniprot.org; accessed December 21, 2016)*.

### Experimental validation of selected UGT1A partners

**Figure 4:**
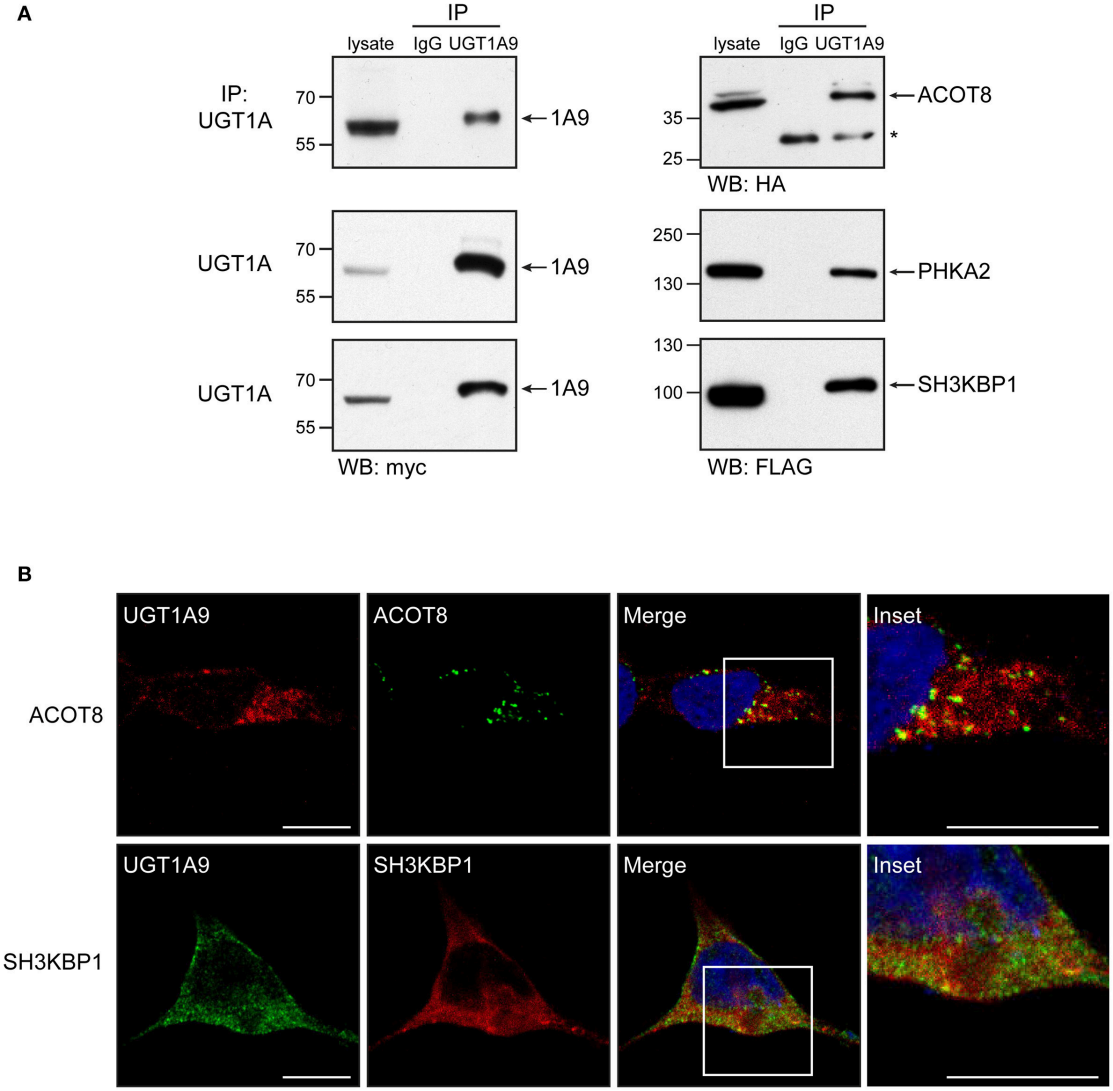
**Validation of selected protein interactions by immunoprecipitation and immunofluorescence in a UGT negative kidney cell model**. **(A)** Immunoprecipitation (IP) of UGT1A9, with purified anti-UGT1A antibodies, was conducted in HEK293-UGT1A9_myc/his transiently transfected with the indicated protein partner. UGT1A9 was immunodetected with anti-myc, whereas protein partners were detected with anti-tag antibodies as specified below immunoblots. Control IPs were conducted with normal rabbit immunoglobulins (IgG). Lysates (IP input) are shown as references. Protein bands denoted by the asterisk are the rabbit IgGs used in IPs. **(B)** Co-localization of UGT1A9 and the protein partners ACOT8 and SH3KBP1/CIN85 assessed by immunofluorescence in HEK293-UGT1A9_myc/his transiently expressing specified partners. Confocal microscope images are representative of three independent experiments. Partial co-localization is detected by yellow labeling in merged images. Insets present enlargements of boxed regions in merged images. Bar = 20 μm.

Using the non-malignant kidney model cell line HEK293 (a UGT negative model) stably expressing a myc/his-tagged UGT1A9 enzyme, selected partnerships with enzymes of bioenergetic cellular pathways were confirmed by a co-IP/immunodetection approach. The peroxisomal acyl-coenzyme A thioesterase ACOT8, involved in fatty acid β-oxidation, and the cytosolic phosphorylase b kinase regulatory subunit A2 (PHKA2), involved in glycogen degradation, were selected based on their significant enrichment in more than one tissue (ACOT8 in kidney, intestine and HT-29; PHKA2 in all 4 matrices). The cytosolic SH3 domain-containing kinase-binding protein 1, also known as cbl-interacting protein of 85 kDa (SH3KBP1/CIN85), an adaptor protein regulating membrane trafficking and receptor signaling, was also chosen as a representative protein partner of the vesicular trafficking pathway, given its identification in the kidney and HT-29 datasets. After transient expression of selected partners as tagged proteins in the kidney cell model stably expressing UGT1A9, each of the candidate partners was specifically enriched by an IP of UGT1A (Figure 4A). Likelihood of a physical interaction of ACOT8 and CIN85 with UGT1A enzymes was further supported by their partial co-localization with UGT1A9 detected by IF and confocal microscopy (Figure 4B).

### Influence of UGT1A on cellular lipid droplets

**Figure 5:**
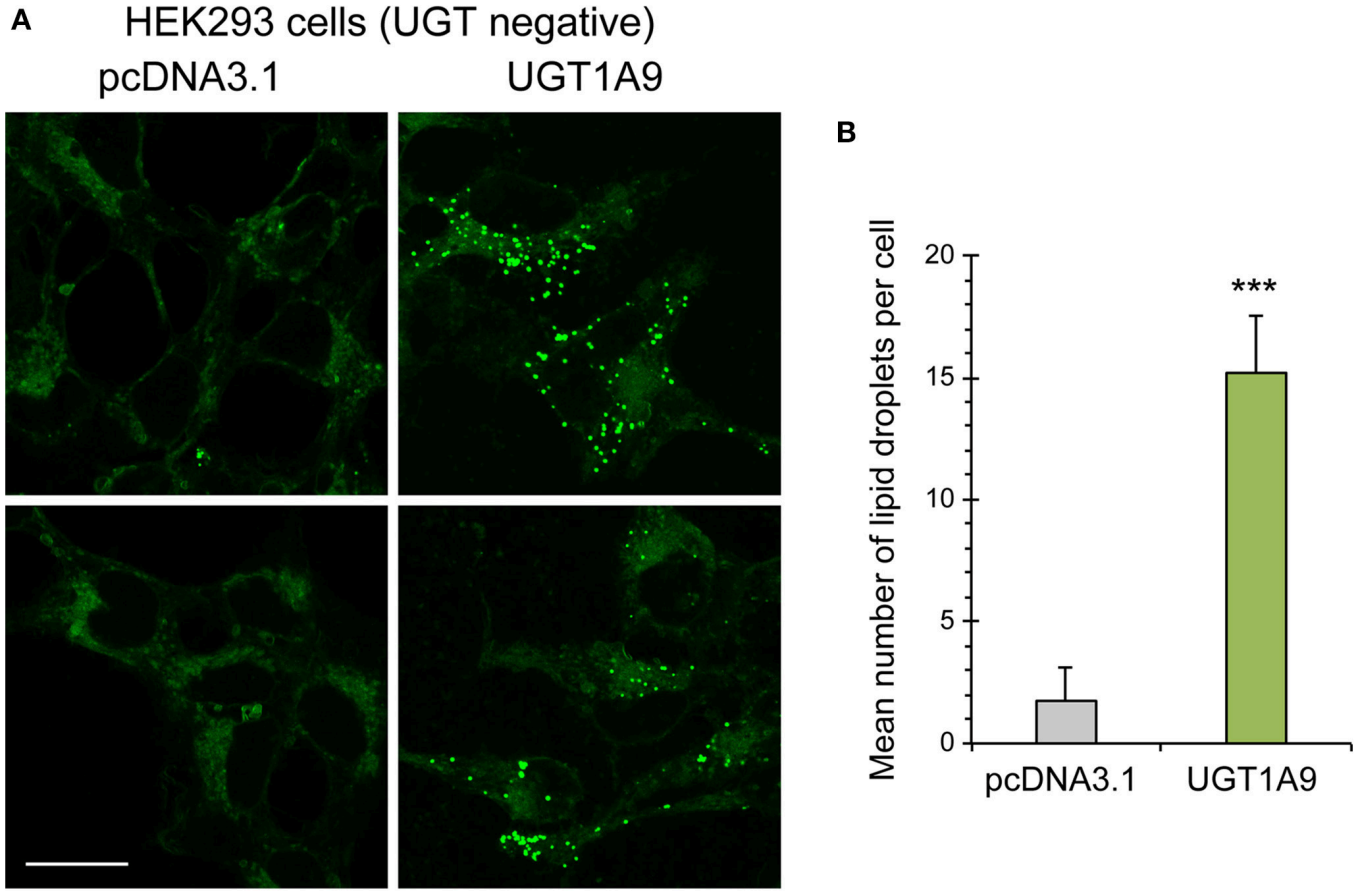
**Accumulation of cellular lipid droplets in UGT1A9 expressing HEK293 cells. (A)** Representative images of lipid droplets (green fluorescence) stained with Nile Red in HEK293-UGT1A9_myc-his or control HEK cells (stably transfected with the empty pcDNA3.1 vector—UGT negative cells). Bar = 20 μm. **(B)** Average number of lipid droplets per cell stably expressing UGT1A9 or control HEK cells. Lipid droplets per cell were counted in at least 140 cells per condition and averaged (*n* = 3 independent experiments).

Pathway enrichment analysis identified several proteins involved in lipid metabolism and suggested a possible functional implication of UGT1A enzymes in this pathway. This was explored by measuring levels of lipid droplet in HEK293 cells stably expressing or not the UGT1A9 enzyme. Lipid droplets, cytoplasmic organelles that constitute a store of neutral lipids such as triacylglycerides, were labeled with Nile Red and counted. This analysis revealed that the number of lipid droplets per cell was significantly higher in UGT1A9-expressing cells relative to control cells (by 7.5-fold, *P* < 0.001), whereas average size and staining intensity of lipid droplets were similar between UGT negative and UGT1A9-expressing HEK293 cells (Figure 5).

## Discussion

Defining protein interaction networks is a key step toward a better understanding of functional crosstalk among cellular pathways. In the current work, we established the endogenous interactome of key metabolic UGT1A enzymes in three relevant human tissues. Data suggest an interplay between UGT1A enzymes regulating the glucuronidation pathway and enzymes involved in multiple cellular energetic pathways, most notably with lipid and glucose/glycogen metabolism. This interactome considerably expands what was known about UGT1A protein interactions in the literature (reviewed by Ishii et al., 2010; Fujiwara et al., 2016) and public databases (3 interactions among UGT1A enzymes reported in STRING database (http://string-db.org/), none in the iRefWEB database (http://wodaklab.org/iRefWeb/), accessed November 9, 2016).

One of our study's strength relies on the use of an unbiased approach targeting endogenous proteins in non-malignant human tissues, as opposed to most studies that used the overexpression of an exogenous tagged protein expressed in a cellular model (Taura et al., 2000; Takeda et al., 2005a,b, 2009; Fujiwara et al., 2007a,b, 2010; Kurkela et al., 2007). In addition, profiles of immunoprecipitated UGT1A enzymes replicated well their known tissue distribution, and spectral peptide counting further reflected the relative abundance of these UGT1A enzymes previously established by mass spectrometry-based multiple reaction monitoring and RNA-sequencing (Fallon et al., 2013a,b; Sato et al., 2014; Margaillan et al., 2015a,b; Tourancheau et al., 2016). Of note, our data support the notion that both UGT1A8 and UGT1A10 enzymes are expressed in the intestine, as peptides unique to each UGT were detected (Figure 2; Supplementary Table 1) (Strassburg et al., 2000; Sato et al., 2014; Fujiwara et al., 2016; Troberg et al., 2016). Moreover, two peptides specific to the UGT1A5 enzyme sequence were detected in the intestine, albeit at low levels (1 spectrum for each peptide) relative to other expressed UGT1As, providing evidence for its intestinal expression at the protein level (Supplementary Figure 4).

We provide unprecedented data on protein-protein interactions within the UGT family, namely between UGT1A and UGT2A3 enzymes and/or UGT2B family members. UGT1A-UGT2 interactions were observed in the liver (UGT2B4 and UGT2B7), in the kidney (UGT2B7) and in the intestine (UGT2B7, UGT2B17, and UGT2A3), and reflect the expression profiles of these UGT2 enzymes (Harbourt et al., 2012; Fallon et al., 2013a,b; Sato et al., 2014; Margaillan et al., 2015a,b; Tourancheau et al., 2016). Our current study offers a representative view of the endogenous UGT1A enzyme interactome in relevant drug metabolizing tissues. Findings are consistent with the interactions between several UGT1A enzymes and UGT2B7 detected in microsomes from liver tissues (Fremont et al., 2005; Fujiwara and Itoh, 2014) and when overexpressed in heterologous cell model systems as tagged proteins (Kurkela et al., 2007; Operaña and Tukey, 2007; Fujiwara et al., 2010; Ishii et al., 2010, 2014; Liu et al., 2016). In addition, other transferases and anti-oxidant PRDX1, PRDX2 and PRDX3 enzymes were also found associated with UGT1A enzymes. The interaction network is also in line with a model favoring detoxifying enzymes acting in a “metabolosome,” i.e. a complex of xenobiotic-metabolizing enzymes and associated transport proteins regulating drug and xenobiotics inactivation and elimination (Taura et al., 2000; Takeda et al., 2005a,b, 2009; Akizawa et al., 2008; Mori et al., 2011; Fujiwara and Itoh, 2014; Ishii et al., 2014; Rouleau et al., 2014; Fujiwara et al., 2016).

The significant number of peroxisomal and mitochondrial enzymes regulating fatty acid β-oxidation identified in protein complexes with UGT1A enzymes hinted toward a potential involvement of UGT1A in regulating lipid metabolism. The higher number of lipid droplets, a reservoir of neutral lipids (such as fatty acids, sterol esters and phospholipids) (Thiam et al., 2013), in the UGT negative kidney cell model HEK293 overexpressing UGT1A9 lends support to this hypothesis. This observation is reminiscent of higher levels of lipid bodies induced by the overexpression of the peroxisomal ACOT8 protein, a confirmed UGT1A protein partner (Ishizuka et al., 2004). A modulation of lipid storage levels by overexpression of the UGT2B7 enzyme was also recently uncovered in breast and pancreatic cancer cell line models (Dates et al., 2015). This potential functional link between UGTs and lipid metabolism is intriguing and may be independent of the glucuronidation of some bioactive lipids previously reported (Turgeon et al., 2003). The underlying mechanism(s) of increased lipid droplets and the potential involvement of protein complexes comprised of UGT1A enzymes thus remain to be addressed and are aspects that fall beyond the scope of this study.

While UGT1A are ER-resident enzymes, their presence in other subcellular compartments such as the mitochondria is suggested by their co-localization with markers of several organelles (Rouleau et al., 2016). An intimate connection between ER, mitochondria, peroxisomes, and lipid droplets is also well recognized (Currie et al., 2013; Schrader et al., 2015). This is consistent with the significant number of peroxisomal and mitochondrial proteins interacting with UGT1A enzymes. Indeed, peroxisomes and lipid droplets are ER-derived substructures, whereas interactions between the ER and mitochondria at the so-called mitochondria-associated ER membranes are gaining recognition as important sites of ER-mitochondria crosstalk where regulation of calcium signaling, lipid transport and tricarboxylic acid cycle take place (Hayashi et al., 2009; Tabak et al., 2013; Lodhi and Semenkovich, 2014; Pol et al., 2014).

The UGT1A interaction network exposes multiple links with enzymes of bioenergetic pathways. Besides lipids, glycogen catabolism as well as glycolytic and tricarboxylic acid cycle pathways may be influenced by the interactions of UGT1A with several subunits of the phosphorylase b and glycolytic/TCA cycle enzymes. It could be envisioned that UGT1A enzymes participate in the regulation of metabolite levels to prevent the toxic impact of excess concentrations of basic constituents, a hypothesis that remains to be addressed. Interestingly, mice with a disrupted *UGT1* gene locus (*UGT1*^−/−^ mice) are short-lived, dying within 1 week of birth. Whereas hyperbilirubinemia induced by UGT1A1 deficiency appears largely responsible for early death, highly perturbed hepatic expression of genes involved in general cellular metabolic function, and notably those of starch, sugar and fatty acid metabolism was also observed in *UGT1*^−/−^ mice, also supporting a contribution of UGT1A enzymes in those metabolic pathways (Nguyen et al., 2008). Rodent cell models from UGT1A-deficient mice or Gunn rats may constitute valuable models to investigate the interplay between UGT1A enzymes and global metabolic pathways.

One of the limitations of this study is that it examines complexes in which UGT1A enzymes reside and it does not provide information on direct interactions of UGT1A with proteins. Approaches such as proximity ligation and fluorescence resonance energy transfer are necessary to move forward with a better understanding of direct protein interactions and the domains involved. It is well documented that UGTs, like numerous metabolic enzymes, homo- and hetero-oligomerize with other UGTs (Fujiwara et al., 2007a; Kurkela et al., 2007; Operaña and Tukey, 2007; Bellemare et al., 2010b). It is therefore conceivable that UGT1A enzymes influence the activity of other metabolic enzymes by direct interactions that could alter the stoichiometry or composition of metabolic protein complexes. In turn, interactions of UGT1A enzymes with other metabolic enzymes may influence the glucuronidation pathway and thus contribute to the variable conjugation rates of individuals. This notion is supported by the altered activity of several UGT1A enzymes by CYP3A4 demonstrated in a cell-based system (Ishii et al., 2014). As well, the antagonistic or stimulatory functions of interactions among UGT1A and UGT2 enzymes, or with alternatively spliced isoforms, are consistent with a potential mode of regulation of UGTs by PPI (Fujiwara et al., 2007a; Bellemare et al., 2010b; Bushey and Lazarus, 2012; Rouleau et al., 2014, 2016; Audet-Delage et al., 2017).

In summary, we established an effective affinity purification method coupled to mass spectrometry for the enrichment and identification of protein complexes interacting with endogenous UGT1A enzymes. We successfully applied this approach to UGT1A enzymes expressed in drug metabolizing tissues and a UGT positive cell model to uncover an interaction map linking glucuronidation enzymes to other metabolic proteins involved in detoxification, as well as in the regulation of bioenergetic molecules (lipids and carbohydrates). Our data also support physical and functional interactions between ER and other subcellular compartments. The crosstalk among cellular metabolic functions exposed in this work warrants future investigations to address the impact of UGT1A-protein interactions on detoxification functions of UGT1A enzymes and of UGT1A enzymes on global metabolic cellular functions.

## Author contributions

Conceptualization: CG; Methodology, MiR, MéR, YAD, CG; Investigation, MéR, YAD, CGB, SD; Formal Analysis, MiR, MéR, YAD, CGB, SD, CG. Writing—Review and Editing, All authors; Visualization, MiR, YAD, CG; Supervision, MiR, CG; Funding Acquisition, CG.

### Conflict of interest statement

The authors declare that the research was conducted in the absence of any commercial or financial relationships that could be construed as a potential conflict of interest.

## Supplementary material

Supplementary Figure S1 **MS identification of human UGT1A in drug metabolizing tissues**.

Supplementary Figure S2 **Confidence interaction scores SAINT vs. FC_B for interaction partners of UGT1A enzymes**.

Supplementary Figure S3 **Overlap of UGT1A enzymes interacting proteins identified in each non-malignant human tissue and in the gastrointestinal cancer model cell line HT-29**.

Supplementary Figure S4 **Identification of UGT1A5 enzyme expression in the intestine**.

Supplementary Table S1 **UGT1A enzymes affinity purified from human tissue S9 fractions**.

Supplementary Table S2 **LC-MS/MS identification of interacting proteins in human tissue S9 fractions**.

Supplementary Table S3 **List of proteins in each group of the Venn diagrams (Figure 1B and Supplementary Figure 2)**.

Supplementary Table S4 **Liver proteomics data**.

Supplementary Table S5 **Kidney proteomics data**.

Supplementary Table S6 **Intestine proteomics data**.

Supplementary Table S7 **HT-29 proteomics data**.

## Abbreviations

AP: affinity purification
UGT: UDP-glucuronosyltransferases
IP: immunoprecipitation
PPIs: protein-protein interactions
UDP-GlcA: Uridine diphospho-glucuronic acid
ER: endoplasmic reticulum
MS: mass spectrometry.

## References

[B1] Akizawa E. Koiwai K. Hayano T. Maezawa S. Matsushita T. Koiwai O. (2008). Direct binding of ligandin to uridine 5'-diphosphate glucuronosyltransferase 1A1. Hepatol. Res. 38, 402–409. 10.1111/j.1872-034X.2007.00285.x 18021224

[B2] Audet-Delage Y. Rouleau M. Rouleau M. Roberge J. Miard S. Picard F.. (2017). Cross-talk between alternatively spliced UGT1A isoforms and colon cancer cell metabolism. Mol. Pharmacol. 91, 1–10. 10.1124/mol.116.106161 28049773

[B3] Bellemare J. Rouleau M. Girard H. Harvey M. Guillemette C. (2010a). Alternatively spliced products of the UGT1A gene interact with the enzymatically active proteins to inhibit glucuronosyltransferase activity *in vitro*. Drug Metab. Dispos. 38, 1785–1789. 10.1124/dmd.110.034835 20610558

[B4] Bellemare J. Rouleau M. Harvey M. Guillemette C. (2010b). Modulation of the human glucuronosyltransferase UGT1A pathway by splice isoform polypeptides is mediated through protein-protein interactions. J. Biol. Chem. 285, 3600–3607. 10.1074/jbc.M109.083139 19996319 PMC2823500

[B5] Bellemare J. Rouleau M. Harvey M. Popa I. Pelletier G. Tetu B.. (2011). Immunohistochemical expression of conjugating UGT1A-derived isoforms in normal and tumoral drug-metabolizing tissues in humans. J. Pathol. 223, 425–435. 10.1002/path.2805 21171088

[B6] Bindea G. Galon J. Mlecnik B. (2013). CluePedia Cytoscape plugin: pathway insights using integrated experimental and *in silico* data. Bioinformatics 29, 661–663. 10.1093/bioinformatics/btt019 23325622 PMC3582273

[B7] Bindea G. Mlecnik B. Hackl H. Charoentong P. Tosolini M. Kirilovsky A.. (2009). ClueGO: a Cytoscape plug-in to decipher functionally grouped gene ontology and pathway annotation networks. Bioinformatics 25, 1091–1093. 10.1093/bioinformatics/btp101 19237447 PMC2666812

[B8] Bolte S. Cordelieres F. P. (2006). A guided tour into subcellular colocalization analysis in light microscopy. J. Microsc. 224, 213–232. 10.1111/j.1365-2818.2006.01706.x 17210054

[B9] Bushey R. T. Lazarus P. (2012). Identification and functional characterization of a novel UDP-glucuronosyltransferase 2A1 splice variant: potential importance in tobacco-related cancer susceptibility. J. Pharmacol. Exp. Ther. 343, 712–724. 10.1124/jpet.112.198770 22984225 PMC3500542

[B10] Choi H. Larsen B. Lin Z. Y. Breitkreutz A. Mellacheruvu D. Fermin D.. (2011). SAINT: probabilistic scoring of affinity purification-mass spectrometry data. Nat. Methods 8, 70–73. 10.1038/nmeth.1541 21131968 PMC3064265

[B11] Costa E. (2006). Hematologically important mutations: bilirubin UDP-glucuronosyltransferase gene mutations in Gilbert and Crigler-Najjar syndromes. Blood Cells Mol. Dis. 36, 77–80. 10.1016/j.bcmd.2005.10.006 16386929

[B12] Currie E. Schulze A. Zechner R. Walther T. C. Farese R. V. Jr. (2013). Cellular fatty acid metabolism and cancer. Cell Metab. 18, 153–161. 10.1016/j.cmet.2013.05.017 23791484 PMC3742569

[B13] Dates C. R. Fahmi T. Pyrek S. J. Yao-Borengasser A. Borowa-Mazgaj B. Bratton S. M.. (2015). Human UDP-Glucuronosyltransferases: effects of altered expression in breast and pancreatic cancer cell lines. Cancer Biol. Ther. 16, 714–723. 10.1080/15384047.2015.1026480 25996841 PMC4622877

[B14] Dluzen D. F. Lazarus P. (2015). MicroRNA regulation of the major drug-metabolizing enzymes and related transcription factors. Drug Metab. Rev. 47, 320–334. 10.3109/03602532.2015.1076438 26300547 PMC6309899

[B15] Fallon J. K. Neubert H. Goosen T. C. Smith P. C. (2013a). Targeted precise quantification of 12 human recombinant uridine-diphosphate glucuronosyl transferase 1A and 2B isoforms using nano-ultra-high-performance liquid chromatography/tandem mass spectrometry with selected reaction monitoring. Drug Metab. Dispos. 41, 2076–2080. 10.1124/dmd.113.053801 24046331 PMC3834135

[B16] Fallon J. K. Neubert H. Hyland R. Goosen T. C. Smith P. C. (2013b). Targeted quantitative proteomics for the analysis of 14 UGT1As and -2Bs in human liver using NanoUPLC-MS/MS with selected reaction monitoring. J. Proteome Res. 12, 4402–4413. 10.1021/pr4004213 23977844

[B17] Fremont J. J. Wang R. W. King C. D. (2005). Coimmunoprecipitation of UDP-glucuronosyltransferase isoforms and cytochrome P450 3A4. Mol. Pharmacol. 67, 260–262. 10.1124/mol.104.006361 15486048

[B18] Fujiwara R. Itoh T. (2014). Extensive protein-protein interactions involving UDP-glucuronosyltransferase (UGT) 2B7 in human liver microsomes. Drug Metab. Pharmacokinet. 29, 259–265. 10.2133/dmpk.DMPK-13-RG-096 24366439

[B19] Fujiwara R. Nakajima M. Oda S. Yamanaka H. Ikushiro S. Sakaki T.. (2010). Interactions between human UDP-glucuronosyltransferase (UGT) 2B7 and UGT1A enzymes. J. Pharm. Sci. 99, 442–454. 10.1002/jps.21830 19475557

[B20] Fujiwara R. Nakajima M. Yamanaka H. Katoh M. Yokoi T. (2007a). Interactions between human UGT1A1, UGT1A4, and UGT1A6 affect their enzymatic activities. Drug Metab. Dispos. 35, 1781–1787. 10.1124/dmd.107.016402 17620344

[B21] Fujiwara R. Nakajima M. Yamanaka H. Nakamura A. Katoh M. Ikushiro S.. (2007b). Effects of coexpression of UGT1A9 on enzymatic activities of human UGT1A isoforms. Drug Metab. Dispos. 35, 747–757. 10.1124/dmd.106.014191 17293379

[B22] Fujiwara R. Yokoi T. Nakajima M. (2016). Structure and protein-protein interactions of human UDP-glucuronosyltransferases. Front. Pharmacol. 7:388. 10.3389/fphar.2016.00388 27822186 PMC5075577

[B23] Guillemette C. Levesque E. Harvey M. Bellemare J. Menard V. (2010). UGT genomic diversity: beyond gene duplication. Drug Metab. Rev. 42, 24–44. 10.3109/03602530903210682 19857043

[B24] Guillemette C. Levesque E. Rouleau M. (2014). Pharmacogenomics of human uridine diphospho-glucuronosyltransferases and clinical implications. Clin. Pharmacol. Ther. 96, 324–339. 10.1038/clpt.2014.126 24922307

[B25] Harbourt D. E. Fallon J. K. Ito S. Baba T. Ritter J. K. Glish G. L.. (2012). Quantification of human uridine-diphosphate glucuronosyl transferase 1A isoforms in liver, intestine, and kidney using nanobore liquid chromatography-tandem mass spectrometry. Anal. Chem. 84, 98–105. 10.1021/ac201704a 22050083 PMC3259189

[B26] Hayashi T. Rizzuto R. Hajnoczky G. Su T. P. (2009). MAM: more than just a housekeeper. Trends Cell Biol. 19, 81–88. 10.1016/j.tcb.2008.12.002 19144519 PMC2750097

[B27] Hu D. G. Meech R. McKinnon R. A. Mackenzie P. I. (2014). Transcriptional regulation of human UDP-glucuronosyltransferase genes. Drug Metab. Rev. 46, 421–458. 10.3109/03602532.2014.973037 25336387

[B28] Hung Y. H. Chan Y. S. Chang Y. S. Lee K. T. Hsu H. P. Yen M. C.. (2014). Fatty acid metabolic enzyme acyl-CoA thioesterase 8 promotes the development of hepatocellular carcinoma. Oncol. Rep. 31, 2797–2803. 10.3892/or.2014.3155 24788990

[B29] Ishii Y. Iwanaga M. Nishimura Y. Takeda S. Ikushiro S. Nagata K.. (2007). Protein-protein interactions between rat hepatic cytochromes P450 (P450s) and UDP-glucuronosyltransferases (UGTs): evidence for the functionally active UGT in P450-UGT complex. Drug Metab. Pharmacokinet. 22, 367–376. 10.2133/dmpk.22.367 17965520

[B30] Ishii Y. Koba H. Kinoshita K. Oizaki T. Iwamoto Y. Takeda S.. (2014). Alteration of the function of the UDP-glucuronosyltransferase 1A subfamily by cytochrome P450 3A4: different susceptibility for UGT isoforms and UGT1A1/7 variants. Drug Metab. Dispos. 42, 229–238. 10.1124/dmd.113.054833 24255116

[B31] Ishii Y. Takeda S. Yamada H. (2010). Modulation of UDP-glucuronosyltransferase activity by protein-protein association. Drug Metab. Rev. 42, 145–158. 10.3109/03602530903208579 19817679

[B32] Ishizuka M. Toyama Y. Watanabe H. Fujiki Y. Takeuchi A. Yamasaki S.. (2004). Overexpression of human acyl-CoA thioesterase upregulates peroxisome biogenesis. Exp. Cell Res. 297, 127–141. 10.1016/j.yexcr.2004.02.029 15194431

[B33] Kurkela M. Patana A. S. Mackenzie P. I. Court M. H. Tate C. G. Hirvonen J.. (2007). Interactions with other human UDP-glucuronosyltransferases attenuate the consequences of the Y485D mutation on the activity and substrate affinity of UGT1A6. Pharmacogenet. Genomics 17, 115–126. 10.1097/FPC.0b013e328011b598 17301691

[B34] Liu Y. Q. Yuan L. M. Gao Z. Z. Xiao Y. S. Sun H. Y. Yu L. S.. (2016). Dimerization of human uridine diphosphate glucuronosyltransferase allozymes 1A1 and 1A9 alters their quercetin glucuronidation activities. Sci. Rep. 6:23763. 10.1038/srep23763 27025983 PMC4837415

[B35] Lodhi I. J. Semenkovich C. F. (2014). Peroxisomes: a nexus for lipid metabolism and cellular signaling. Cell Metab. 19, 380–392. 10.1016/j.cmet.2014.01.002 24508507 PMC3951609

[B36] Margaillan G. Rouleau M. Fallon J. K. Caron P. Villeneuve L. Turcotte V.. (2015a). Quantitative profiling of human renal UDP-glucuronosyltransferases and glucuronidation activity: a comparison of normal and tumoral kidney tissues. Drug Metab. Dispos. 43, 611–619. 10.1124/dmd.114.062877 25650382 PMC4366751

[B37] Margaillan G. Rouleau M. Klein K. Fallon J. K. Caron P. Villeneuve L.. (2015b). Multiplexed targeted quantitative proteomics predicts hepatic glucuronidation potential. Drug Metab. Dispos. 43, 1331–1335. 10.1124/dmd.115.065391 26076694 PMC4538857

[B38] Mellacheruvu D. Wright Z. Couzens A. L. Lambert J. P. St-Denis N. A. Li T.. (2013). The CRAPome: a contaminant repository for affinity purification-mass spectrometry data. Nat. Methods 10, 730–736. 10.1038/nmeth.2557 23921808 PMC3773500

[B39] Ménard V. Collin P. Margaillan G. Guillemette C. (2013). Modulation of the UGT2B7 enzyme activity by C-terminally truncated proteins derived from alternative splicing. Drug Metab. Dispos. 41, 2197–2205. 10.1124/dmd.113.053876 24088326

[B40] Mori Y. Kiyonaka S. Kanai Y. (2011). Transportsomes and channelsomes: are they functional units for physiological responses? Channels 5, 387–390. 10.4161/chan.5.5.16466 21849819

[B41] Nguyen N. Bonzo J. A. Chen S. Chouinard S. Kelner M. J. Hardiman G.. (2008). Disruption of the ugt1 locus in mice resembles human Crigler-Najjar type I disease. J. Biol. Chem. 283, 7901–7911. 10.1074/jbc.M709244200 18180294

[B42] Operaña T. N. Tukey R. H. (2007). Oligomerization of the UDP-glucuronosyltransferase 1A proteins: homo- and heterodimerization analysis by fluorescence resonance energy transfer and co-immunoprecipitation. J. Biol. Chem. 282, 4821–4829. 10.1074/jbc.M609417200 17179145

[B43] Pol A. Gross S. P. Parton R. G. (2014). Review: biogenesis of the multifunctional lipid droplet: lipids, proteins, and sites. J. Cell Biol. 204, 635–646. 10.1083/jcb.201311051 24590170 PMC3941045

[B44] Ramírez J. Ratain M. J. Innocenti F. (2010). Uridine 5′-diphospho-glucuronosyltransferase genetic polymorphisms and response to cancer chemotherapy. Future Oncol. 6, 563–585. 10.2217/fon.10.17 20373870 PMC3102300

[B45] Rouleau M. Roberge J. Bellemare J. Guillemette C. (2014). Dual roles for splice variants of the glucuronidation pathway as regulators of cellular metabolism. Mol. Pharmacol. 85, 29–36. 10.1124/mol.113.089227 24141015

[B46] Rouleau M. Tourancheau A. Girard-Bock C. Villeneuve L. Vaucher J. Duperre A. M.. (2016). Divergent expression and metabolic functions of human glucuronosyltransferases through alternative splicing. Cell Rep. 17, 114–124. 10.1016/j.celrep.2016.08.077 27681425

[B47] Rowland A. Miners J. O. Mackenzie P. I. (2013). The UDP-glucuronosyltransferases: their role in drug metabolism and detoxification. Int. J. Biochem. Cell Biol. 45, 1121–1132. 10.1016/j.biocel.2013.02.019 23500526

[B48] Ruan H. B. Han X. Li M. D. Singh J. P. Qian K. Azarhoush S.. (2012). O-GlcNAc transferase/host cell factor C1 complex regulates gluconeogenesis by modulating PGC-1alpha stability. Cell Metab. 16, 226–237. 10.1016/j.cmet.2012.07.006 22883232 PMC3480732

[B49] Sato Y. Nagata M. Tetsuka K. Tamura K. Miyashita A. Kawamura A.. (2014). Optimized methods for targeted peptide-based quantification of human uridine 5'-diphosphate-glucuronosyltransferases in biological specimens using liquid chromatography-tandem mass spectrometry. Drug Metab. Dispos. 42, 885–889. 10.1124/dmd.113.056291 24595681

[B50] Savas J. N. Stein B. D. Wu C. C. Yates J. R. III. (2011). Mass spectrometry accelerates membrane protein analysis. Trends Biochem. Sci. 36, 388–396. 10.1016/j.tibs.2011.04.005 21616670 PMC3222592

[B51] Schrader M. Godinho L. F. Costello J. L. Islinger M. (2015). The different facets of organelle interplay-an overview of organelle interactions. Front Cell Dev Biol 3:56. 10.3389/fcell.2015.00056 26442263 PMC4585249

[B52] Schroeder B. Weller S. G. Chen J. Billadeau D. McNiven M. A. (2010). A Dyn2-CIN85 complex mediates degradative traffic of the EGFR by regulation of late endosomal budding. EMBO J. 29, 3039–3053. 10.1038/emboj.2010.190 20711168 PMC2944065

[B53] Strassburg C. P. Kneip S. Topp J. Obermayer-Straub P. Barut A. Tukey R. H.. (2000). Polymorphic gene regulation and interindividual variation of UDP-glucuronosyltransferase activity in human small intestine. J. Biol. Chem. 275, 36164–36171. 10.1074/jbc.M002180200 10748067

[B54] Tabak H. F. Braakman I. van der Zand A. (2013). Peroxisome formation and maintenance are dependent on the endoplasmic reticulum. Annu. Rev. Biochem. 82, 723–744. 10.1146/annurev-biochem-081111-125123 23414306

[B55] Takeda S. Ishii Y. Iwanaga M. Mackenzie P. I. Nagata K. Yamazoe Y.. (2005a). Modulation of UDP-glucuronosyltransferase function by cytochrome P450: evidence for the alteration of UGT2B7-catalyzed glucuronidation of morphine by CYP3A4. Mol. Pharmacol. 67, 665–672. 10.1124/mol.104.007641 15611481

[B56] Takeda S. Ishii Y. Iwanaga M. Nurrochmad A. Ito Y. Mackenzie P. I.. (2009). Interaction of cytochrome P450 3A4 and UDP-glucuronosyltransferase 2B7: evidence for protein-protein association and possible involvement of CYP3A4 J-helix in the interaction. Mol. Pharmacol. 75, 956–964. 10.1124/mol.108.052001 19158361

[B57] Takeda S. Ishii Y. Mackenzie P. I. Nagata K. Yamazoe Y. Oguri K.. (2005b). Modulation of UDP-glucuronosyltransferase 2B7 function by cytochrome P450s *in vitro*: differential effects of CYP1A2, CYP2C9 and CYP3A4. Biol. Pharm. Bull. 28, 2026–2027. 10.1248/bpb.28.2026 16204972

[B58] Taura K. I. Yamada H. Hagino Y. Ishii Y. Mori M. A. Oguri K. (2000). Interaction between cytochrome P450 and other drug-metabolizing enzymes: evidence for an association of CYP1A1 with microsomal epoxide hydrolase and UDP-glucuronosyltransferase. Biochem. Biophys. Res. Commun. 273, 1048–1052. 10.1006/bbrc.2000.3076 10891369

[B59] Thiam A. R. Farese R. V. Jr. Walther T. C. (2013). The biophysics and cell biology of lipid droplets. Nat. Rev. Mol. Cell Biol. 14, 775–786. 10.1038/nrm3699 24220094 PMC4526153

[B60] Tourancheau A. Margaillan G. Rouleau M. Gilbert I. Villeneuve L. Levesque E.. (2016). Unravelling the transcriptomic landscape of the major phase II UDP-glucuronosyltransferase drug metabolizing pathway using targeted RNA sequencing. Pharmacogenomics J. 16, 60–70. 10.1038/tpj.2015.20 25869014

[B61] Troberg J. Jarvinen E. Ge G. B. Yang L. Finel M. (2016). UGT1A10 is a high activity and important extrahepatic enzyme: why has its role in intestinal glucuronidation been frequently underestimated? Mol. Pharm. 10.1021/acs.molpharmaceut.6b00852. [Epub ahead of print]. 27966992

[B62] Turgeon D. Chouinard S. Belanger P. Picard S. Labbe J. F. Borgeat P.. (2003). Glucuronidation of arachidonic and linoleic acid metabolites by human UDP-glucuronosyltransferases. J. Lipid Res. 44, 1182–1191. 10.1194/jlr.M300010-JLR200 12639971

