# Supplementary material for: Endogenous Protein Interactome of Human UDP-Glucuronosyltransferases Exposed by Untargeted Proteomics
Source: Front Pharmacol. 2017 Feb 3;8:23. doi: 10.3389/fphar.2017.00023 (PMC5290407; doi:10.3389/fphar.2017.00023)
Supplement: Supplementary Figure S1 — MS identification of human UGT1A in drug metabolizing tissues. [file Image1.PDF]

Unique to a UGT1A - Common to 1A3,1A4 and/or 1A5 – Common to 1A8,1A10 – Common to all UGT1A

```
P22309|UGT1A1_HUMAN    MAVESQGG-RPLVLGLLLCVLGPVVSHAGKILLIPVDGSHWLSMLGAIQQLQQRGHEIVV 59
P35503|UGT1A3_HUMAN    MATGLQVPLPWLATGLLLLLSVQPWAESGKVLVVPIDGSHWLSMREVLRELHARGHQAVV 60
P22310|UGT1A4_HUMAN    MARGLQVPLPRLATGLLLLLSVQPWAESGKVLVVPTDGSPWLSMREALRELHARGHQAVV 60
P35504|UGT1A5_HUMAN    MATGLQVPLPQLATGLLLLLSVQPWAESGKVLVVPTDGSHWLSMREALRDLHARGHQVVV 60
P19224|UGT1A6_HUMAN    MAC-LLRSFQRISAGVFFLA-LWGMVVGDKLLVVPQDGSHWLSMKDIVEVLSDRGHEIVV 58
Q9HAW7|UGT1A7_HUMAN    MARAGWTGLLPL---YVCLLLTCGFAKAGKLLVVPMDGSHWFTMQSVVEKLILRGHEVVV 57
Q9HAW9|UGT1A8_HUMAN    MARTGWTSPIPL---CVSLLLTCGFAEAGKLLVVPMDGSHWFTMQSVVEKLILRGHEVVV 57
O60656|UGT1A9_HUMAN    MACTGWTSPLPL---CVCLLLTCGFAEAGKLLVVPMDGSHWFTMRSVVEKLILRGHEVVV 57
Q9HAW8|UGT1A10_HUMAN   MARAGWTSPVPL---CVCLLLTCGFAEAGKLLVVPMDGSHWFTMQSVVEKLILRGHEVVV 57
                       **          :    .          .  *:*::* *** *::*   :. *   ***: **

P22309|UGT1A1_HUMAN    LAPDASLYIRDGAFYTLKTYPVPFQREDVKESFVSLGHNVFENDSFLQRVIKTYKKIKKD 119
P35503|UGT1A3_HUMAN    LTPEVNMHIKEENFFTLTTYAISWTQDEFDRHVLGHTQLYFETEHFLKKFFRSMAMLNNM 120
P22310|UGT1A4_HUMAN    LTPEVNMHIKEEKFFTLTAYAVPWTQKEFDRVTLGYTQGFFETEHLLKRYSRSMAIMNNV 120
P35504|UGT1A5_HUMAN    LTLEVNMYIKEENFFTLTTYAISWTQDEFDRLLLGHTQSFFETEHLLMKFSRRMAIMNNM 120
P19224|UGT1A6_HUMAN    VVPEVNLLLKESKYYTRKIYPVPYDQEELKNRYQSFGNNHFAERSFLTAPQTEYRNNMIV 118
Q9HAW7|UGT1A7_HUMAN    VMPEVSWQLGRSLNCTVKTYSTSYTLEDQDREFMVFADARWTAPLRS-AFSLLTSSSNGI 116
Q9HAW9|UGT1A8_HUMAN    VMPEVSWQLGKSLNCTVKTYSTSYTLEDLDREFMDFADAQWKAQVRS-LFSLFLSSSNGF 116
O60656|UGT1A9_HUMAN    VMPEVSWQLGRSLNCTVKTYSTSYTLEDLDREFKAFAHAQWKAQVRS-IYSLLMGSYNDI 116
Q9HAW8|UGT1A10_HUMAN   VMPEVSWQLERSLNCTVKTYSTSYTLEDQNREFMVFAHAQWKAQAQS-IFSLLMSSSSGF 116
                       :  :..  :      * . *    :  .: ..       .  :

P22309|UGT1A1_HUMAN    SAMLLSGCSHLLHNKELMASLAESSFDVMLTDPFLPCSPIVAQYLSLPTVFFLHALPCSL 179
P35503|UGT1A3_HUMAN    SLVYHRSCVELLHNEALIRHLNATSFDVVLTDPVNLCAAVLAKYLSIPTVFFLRNIPCDL 180
P22310|UGT1A4_HUMAN    SLALHRCCVELLHNEALIRHLNATSFDVVLTDPVNLCGAVLAKYLSIPAVFFWRYIPCDL 180
P35504|UGT1A5_HUMAN    SLIIHRSCVELLHNEALIRHLHATSFDVVLTDPFHLCAAVLAKYLSIPAVFFLRNIPCDL 180
P19224|UGT1A6_HUMAN    IGLYFINCQSLLQDRDTLNFFKESKFDALFTDPALPCGVILAEYLGLPSVYLFRGFPCSL 178
Q9HAW7|UGT1A7_HUMAN    FDLFFSNCRSLFNDRKLVEYLKESCFDAVFLDPFDACGLIVAKYFSLPSVVFARGIFCHY 176
Q9HAW9|UGT1A8_HUMAN    FNLFFSHCRSLFNDRKLVEYLKESSFDAVFLDPFDACGLIVAKYFSLPSVVFARGIACHY 176
O60656|UGT1A9_HUMAN    FDLFFSNCRSLFKDKKLVEYLKESSFDAVFLDPFDNCGLIVAKYFSLPSVVFARGILCHY 176
Q9HAW8|UGT1A10_HUMAN   LDLFFSHCRSLFNDRKLVEYLKESSFDAVFLDPFDTCGLIVAKYFSLPSVVFTRGIFCHH 176
                              *  *:.:.  :  :  : **.:: **   *. ::*:*:.:*:* : : : *

P22309|UGT1A1_HUMAN    EFEATQCPNPFSYVPRPLSSHSDHMTFLQRVKNMLIAFSQNFLCDVVYSPYATLASEFLQ 239
P35503|UGT1A3_HUMAN    DFKGTQCPNPSSYIPRLLTTNSDHMTFMQRVKNMLYPLALSYICHAFSAPYASLASELFQ 240
P22310|UGT1A4_HUMAN    DFKGTQCPNPSSYIPKLLTTNSDHMTFLQRVKNMLYPLALSYICHTFSAPYASLASELFQ 240
P35504|UGT1A5_HUMAN    DFKGTQCPNPSSYIPRLLTTNSDHMTFLQRVKNMLYPLALSYLCHAVSAPYASLASELFQ 240
P19224|UGT1A6_HUMAN    EHTFSRSPDPVSYIPRCYTKFSDHMTFSQRVANFLVNLLEPYLFYCLFSKYEELASAVLK 238
Q9HAW7|UGT1A7_HUMAN    LEEGAQCPAPLSYVPRLLLGFSDAMTFKERVWNHIMHLEEHLFCPYFFKNVLEIASEILQ 236
Q9HAW9|UGT1A8_HUMAN    LEEGAQCPAPLSYVPRILLGFSDAMTFKERVRNHIMHLEEHLFCQYFSKNALEIASEILQ 236
O60656|UGT1A9_HUMAN    LEEGAQCPAPLSYVPRILLGFSDAMTFKERVRNHIMHLEEHLLCHRFFKNALEIASEILQ 236
Q9HAW8|UGT1A10_HUMAN   LEEGAQCPAPLSYVPNDLLGFSDAMTFKERVWNHIVHLEDHLFCQYLFRNALEIASEILQ 236
                           ::.* * **:*.     ** *** :** * :  :    :   .      :** .::

P22309|UGT1A1_HUMAN    REVTVQDLLSSASVWLFRSDFVKDYPRPIMPNMVFVGGINCLHQNPLSQEFEAYINASGE 299
P35503|UGT1A3_HUMAN    REVSVVDILSHASVWLFRGDFVMDYPRPIMPNMVFIGGINCANRKPLSQEFEAYINASGE 300
P22310|UGT1A4_HUMAN    REVSVVDLVSYASVWLFRGDFVMDYPRPIMPNMVFIGGINCANGKPLSQEFEAYINASGE 300
P35504|UGT1A5_HUMAN    REVSVVDLVSHASVWLFRGDFVMDYPRPIMPNMVFIGGINCANGKPLSQEFEAYINASGE 300
P19224|UGT1A6_HUMAN    RDVDIITLYQKVSVWLLRYDFVLEYPRPVMPNMVFIGGINCKKRKDLSQEFEAYINASGE 298
Q9HAW7|UGT1A7_HUMAN    TPVTAYDLYSHTSIWLLRTDFVLEYPKPVMPNMIFIGGINCHQGKPVPMEFEAYINASGE 296
Q9HAW9|UGT1A8_HUMAN    TPVTAYDLYSHTSIWLLRTDFVLDYPKPVMPNMIFIGGINCHQGKPLPMEFEAYINASGE 296
O60656|UGT1A9_HUMAN    TPVTEYDLYSHTSIWLLRTDFVLDYPKPVMPNMIFIGGINCHQGKPLPMEFEAYINASGE 296
Q9HAW8|UGT1A10_HUMAN   TPVTAYDLYSHTSIWLLRTDFVLDYPKPVMPNMIFIGGINCHQGKPLPMEFEAYINASGE 296
                         *    :  . .*:**:* *** :**:*:****:*:*****  . : :  ***********
```

```
P22309|UGT1A1_HUMAN    HGIVVFSLGSMVSEIPEKKAMAIADALGKIPQTVLWRYTGTRPSNLANNTILVKWLPQND 359
P35503|UGT1A3_HUMAN    HGIVVFSLGSMVSEIPEKKAMAIADALGKIPQTVLWRYTGTRPSNLANNTILVKWLPQND 360
P22310|UGT1A4_HUMAN    HGIVVFSLGSMVSEIPEKKAMAIADALGKIPQTVLWRYTGTRPSNLANNTILVKWLPQND 360
P35504|UGT1A5_HUMAN    HGIVVFSLGSMVSEIPEKKAMAIADALGKIPQTVLWRYTGTRPSNLANNTILVKWLPQND 360
P19224|UGT1A6_HUMAN    HGIVVFSLGSMVSEIPEKKAMAIADALGKIPQTVLWRYTGTRPSNLANNTILVKWLPQND 358
Q9HAW7|UGT1A7_HUMAN    HGIVVFSLGSMVSEIPEKKAMAIADALGKIPQTVLWRYTGTRPSNLANNTILVKWLPQND 356
Q9HAW9|UGT1A8_HUMAN    HGIVVFSLGSMVSEIPEKKAMAIADALGKIPQTVLWRYTGTRPSNLANNTILVKWLPQND 356
O60656|UGT1A9_HUMAN    HGIVVFSLGSMVSEIPEKKAMAIADALGKIPQTVLWRYTGTRPSNLANNTILVKWLPQND 356
Q9HAW8|UGT1A10_HUMAN   HGIVVFSLGSMVSEIPEKKAMAIADALGKIPQTVLWRYTGTRPSNLANNTILVKWLPQND 356
                       ************************************************************

P22309|UGT1A1_HUMAN    LLGHPMTRAFITHAGSHGVYESICNGVPMVMMPLFGDQMDNAKRMETKGAGVTLNVLEMT 419
P35503|UGT1A3_HUMAN    LLGHPMTRAFITHAGSHGVYESICNGVPMVMMPLFGDQMDNAKRMETKGAGVTLNVLEMT 420
P22310|UGT1A4_HUMAN    LLGHPMTRAFITHAGSHGVYESICNGVPMVMMPLFGDQMDNAKRMETKGAGVTLNVLEMT 420
P35504|UGT1A5_HUMAN    LLGHPMTRAFITHAGSHGVYESICNGVPMVMMPLFGDQMDNAKRMETKGAGVTLNVLEMT 420
P19224|UGT1A6_HUMAN    LLGHPMTRAFITHAGSHGVYESICNGVPMVMMPLFGDQMDNAKRMETKGAGVTLNVLEMT 418
Q9HAW7|UGT1A7_HUMAN    LLGHPMTRAFITHAGSHGVYESICNGVPMVMMPLFGDQMDNAKRMETKGAGVTLNVLEMT 416
Q9HAW9|UGT1A8_HUMAN    LLGHPMTRAFITHAGSHGVYESICNGVPMVMMPLFGDQMDNAKRMETKGAGVTLNVLEMT 416
O60656|UGT1A9_HUMAN    LLGHPMTRAFITHAGSHGVYESICNGVPMVMMPLFGDQMDNAKRMETKGAGVTLNVLEMT 416
Q9HAW8|UGT1A10_HUMAN   LLGHPMTRAFITHAGSHGVYESICNGVPMVMMPLFGDQMDNAKRMETKGAGVTLNVLEMT 416
                       ************************************************************

P22309|UGT1A1_HUMAN    SEDLENALKAVINDKSYKENIMRLSSLHKDRPVEPLDLAVFWVEFVMRHKGAPHLRPAAH 479
P35503|UGT1A3_HUMAN    SEDLENALKAVINDKSYKENIMRLSSLHKDRPVEPLDLAVFWVEFVMRHKGAPHLRPAAH 480
P22310|UGT1A4_HUMAN    SEDLENALKAVINDKSYKENIMRLSSLHKDRPVEPLDLAVFWVEFVMRHKGAPHLRPAAH 480
P35504|UGT1A5_HUMAN    SEDLENALKAVINDKSYKENIMRLSSLHKDRPVEPLDLAVFWVEFVMRHKGAPHLRPAAH 480
P19224|UGT1A6_HUMAN    SEDLENALKAVINDKSYKENIMRLSSLHKDRPVEPLDLAVFWVEFVMRHKGAPHLRPAAH 478
Q9HAW7|UGT1A7_HUMAN    SEDLENALKAVINDKSYKENIMRLSSLHKDRPVEPLDLAVFWVEFVMRHKGAPHLRPAAH 476
Q9HAW9|UGT1A8_HUMAN    SEDLENALKAVINDKSYKENIMRLSSLHKDRPVEPLDLAVFWVEFVMRHKGAPHLRPAAH 476
O60656|UGT1A9_HUMAN    SEDLENALKAVINDKSYKENIMRLSSLHKDRPVEPLDLAVFWVEFVMRHKGAPHLRPAAH 476
Q9HAW8|UGT1A10_HUMAN   SEDLENALKAVINDKSYKENIMRLSSLHKDRPVEPLDLAVFWVEFVMRHKGAPHLRPAAH 476
                       ************************************************************

P22309|UGT1A1_HUMAN    DLTWYQYHSLDVIGFLLAVVLTVAFITFKCCAYGYRKCLGKKGRVKKAHKSKTH 533
P35503|UGT1A3_HUMAN    DLTWYQYHSLDVIGFLLAVVLTVAFITFKCCAYGYRKCLGKKGRVKKAHKSKTH 534
P22310|UGT1A4_HUMAN    DLTWYQYHSLDVIGFLLAVVLTVAFITFKCCAYGYRKCLGKKGRVKKAHKSKTH 534
P35504|UGT1A5_HUMAN    DLTWYQYHSLDVIGFLLAVVLTVAFITFKCCAYGYRKCLGKKGRVKKAHKSKTH 534
P19224|UGT1A6_HUMAN    DLTWYQYHSLDVIGFLLAVVLTVAFITFKCCAYGYRKCLGKKGRVKKAHKSKTH 532
Q9HAW7|UGT1A7_HUMAN    DLTWYQYHSLDVIGFLLAVVLTVAFITFKCCAYGYRKCLGKKGRVKKAHKSKTH 530
Q9HAW9|UGT1A8_HUMAN    DLTWYQYHSLDVIGFLLAVVLTVAFITFKCCAYGYRKCLGKKGRVKKAHKSKTH 530
O60656|UGT1A9_HUMAN    DLTWYQYHSLDVIGFLLAVVLTVAFITFKCCAYGYRKCLGKKGRVKKAHKSKTH 530
Q9HAW8|UGT1A10_HUMAN   DLTWYQYHSLDVIGFLLAVVLTVAFITFKCCAYGYRKCLGKKGRVKKAHKSKTH 530
                       ******************************************************
```

Supplementary Figure 1. MS identification of human UGT1A in drug metabolizing tissues. Peptides identified for each UGT1A enzyme in all tissues are highlighted on the multiple sequence alignment. Accession number for each UGT1A sequence is provided in the sequence name. Peptides highlighted in yellow are unique to a given UGT1A. Peptides highlighted in blue are common to UGT1A3, UGT1A4 and/or UGT1A5; note that the tryptic peptide at position 197-210 may also be common to UGT1A3 because methionine (M) 208 in UGT1A3 can also be a leucine (L) (documented genetic variant https://www.pharmacogenomics.pha.ulaval.ca/wp-content/uploads/2015/04/SNP-UGT1A3.htm). Peptides highlighted in green are common to UGT1A8 and UGT1A10. Peptides highlighted in grey are common to all UGT1As. Underlined lysines (K) and arginines (R) indicate position of tryptic cleavage. Multiple sequence alignment was created with CLUSTAL O (1.2.1). Conserved residues are marked by an asterisk (*); conservative substitutions by a colon (:); semi-conservative substitutions by a period (.).
