# Supplementary material for: Endogenous Protein Interactome of Human UDP-Glucuronosyltransferases Exposed by Untargeted Proteomics
Source: Front Pharmacol. 2017 Feb 3;8:23. doi: 10.3389/fphar.2017.00023 (PMC5290407; doi:10.3389/fphar.2017.00023)
Supplement: Supplementary Figure S2 — Confidence interaction scores SAINT vs. FC_B for interaction partners of UGT1A enzymes. [file Image2.PDF]

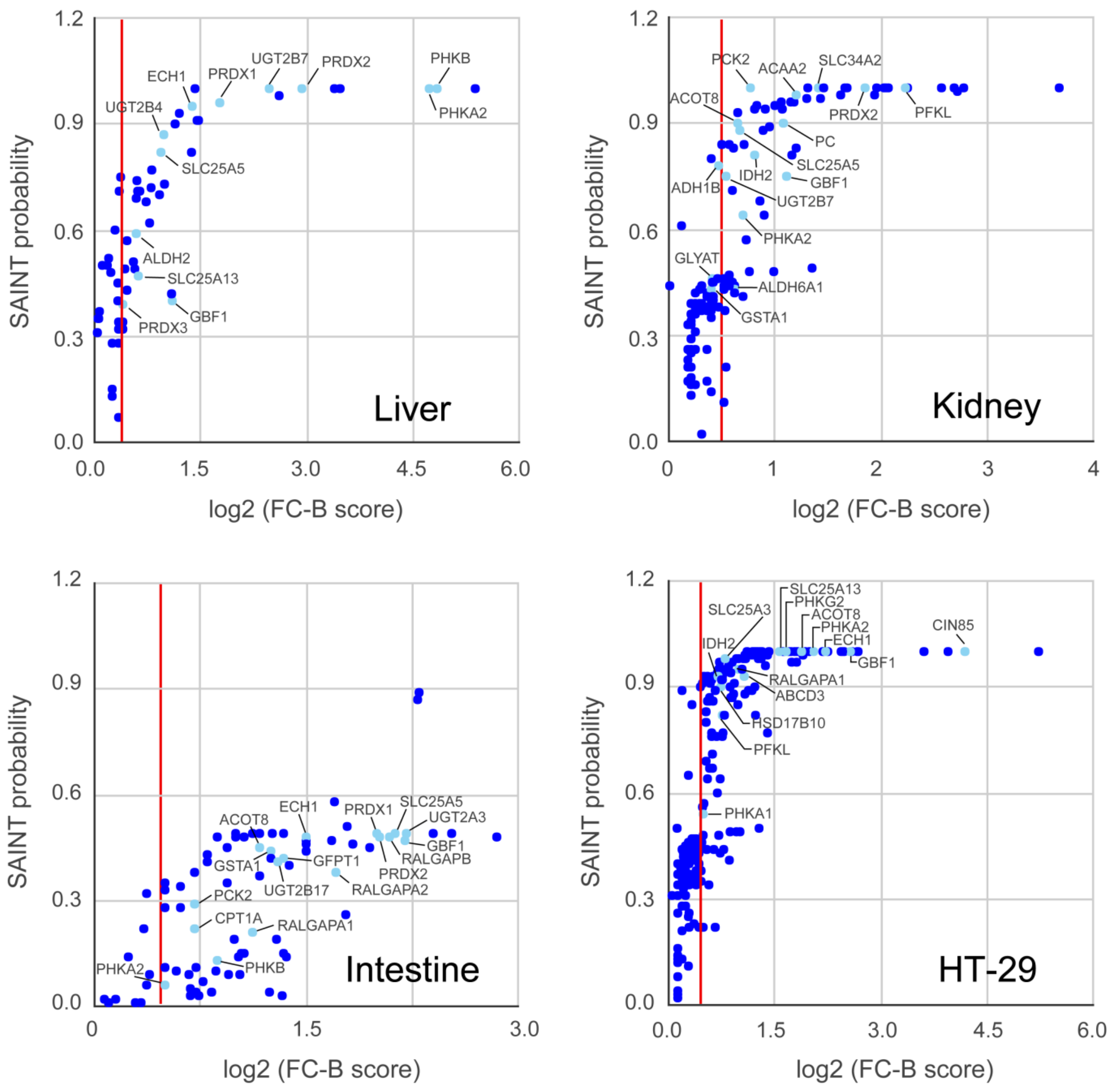

Supplementary Figure 2. Confidence interaction scores SAINT vs FC_B for interaction partners of UGT1A enzymes in non-malignant liver, kidney, intestine tissues and the HT-29 UGT positive cell model. The red line indicates the position of the selected FC_B threshold (1.42). Labeled dots are shown in light blue to facilitate visualization.
