# Supplementary material for: Endogenous Protein Interactome of Human UDP-Glucuronosyltransferases Exposed by Untargeted Proteomics
Source: Front Pharmacol. 2017 Feb 3;8:23. doi: 10.3389/fphar.2017.00023 (PMC5290407; doi:10.3389/fphar.2017.00023)
Supplement: Supplementary Figure S3 — Overlap of UGT1A enzymes interacting proteins identified in each non-malignant human tissue and in the gastrointestinal cancer model cell line HT-29. [file Image3.PDF]

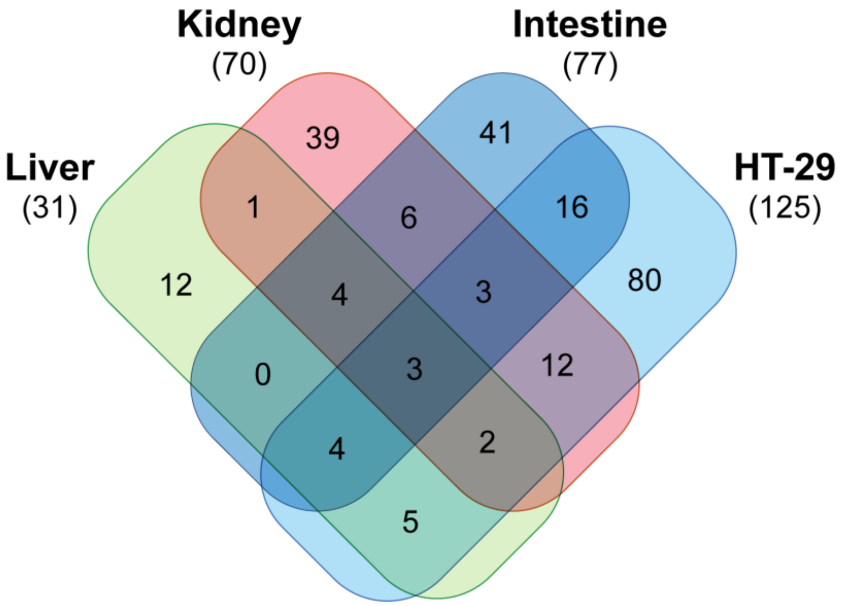

Supplementary Figure 3. Overlap of UGT1A enzymes interacting proteins identified in each non-malignant human tissue and in the gastrointestinal cancer model cell line HT-29. List of proteins in each group is given in Table S3.
