# Supplementary material for: Endogenous Protein Interactome of Human UDP-Glucuronosyltransferases Exposed by Untargeted Proteomics
Source: Front Pharmacol. 2017 Feb 3;8:23. doi: 10.3389/fphar.2017.00023 (PMC5290407; doi:10.3389/fphar.2017.00023)
Supplement: Supplementary Figure S4 — Identification of UGT1A5 enzyme expression in the intestine. [file Image4.PDF]

A Peptide VLVVPTDGSHWLSMR

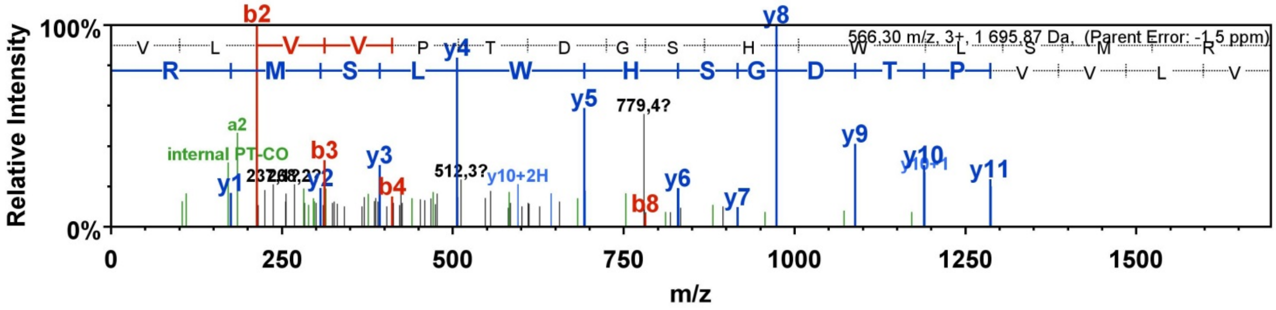

UGT1A5	31-VLVVPTDGSHWLSMR-45
UGT1A4	31-VLVVPTDGSPWLSMR-45
UGT1A3	31-VLVVPIDGSHWLSMR-45

B Peptide YLSIPAVFFLR

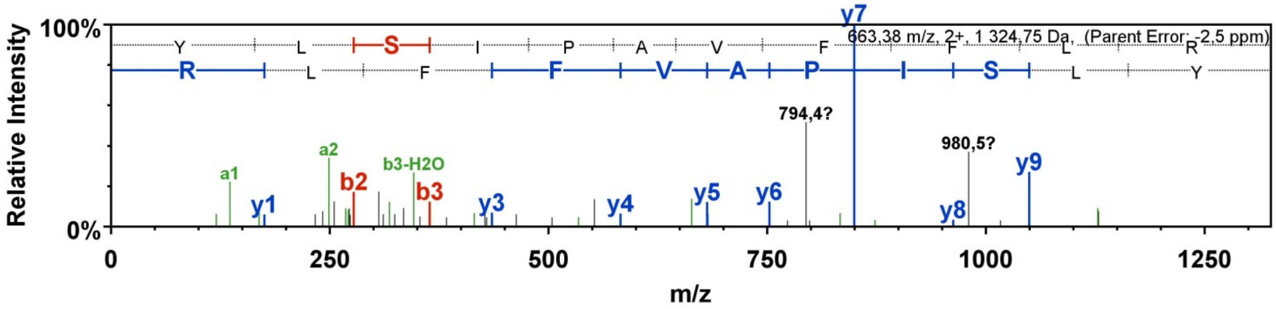

UGT1A5	164-YLSIPAVFFLR-174
UGT1A4	164-YLSIPAVFFWR-174
UGT1A3	164-YLSIPTVFFLR-174

Supplementary Figure 4. Identification of UGT1A5 enzyme expression in the intestine. Two peptides unique to the sequence of UGT1A5 were identified in AP-MS of UGT1A enzymes in the intestine. Fragmentation pattern for each peptide are shown (1 spectrum was obtained per peptide). Multiple alignments of UGT1A3, UGT1A4 and UGT1A5 are shown for the identified UGT1A5 sequences and divergent amino acids are in red.
