# Supplementary material for: Endogenous Protein Interactome of Human UDP-Glucuronosyltransferases Exposed by Untargeted Proteomics
Source: Front Pharmacol. 2017 Feb 3;8:23. doi: 10.3389/fphar.2017.00023 (PMC5290407; doi:10.3389/fphar.2017.00023)
Supplement: Supplementary Table S3 — List of proteins in each group of the Venn diagrams (Figure 1B and Supplementary Figure 2). [file Table3.PDF]

Supplementary Table 3: Proteins classification according to AP-MS identification in matrices (see Fig. S2)

**Group**	**Number of proteins**	**Gene names**
Liver, Kidney, Intestine, HT-29	3	GBF1, MYH9, PHKA2
Liver, Kidney, Intestine	4	RPL7A, PRDX1, PRDX2, SLC25A5
Liver, Kidney, HT-29	2	SRSF7, HSPA1A
Liver, Intestine, HT-29	4	HSPA5, TUFM, ECH1, PHKB
Kidney, Intestine, HT-29	3	RPL10, ATP5A1, ACOT8
Liver, Kidney	1	UGT2B7*
Liver, HT-29	5	PPL, SLC25A13, CCDC50, NAMPT, PHKG2
Kidney, Intestine	6	RPL19, PCK2, RPS3, RPS9, EEF1A1, HNRNPC
Kidney, HT-29	12	PFKL, MYCBP2, RPS2, TUBA4A, HNRNPM, SRSF1, IDH2, SRRM2, MYL6, ATP5C1, MBNL1, SRSF6
Intestine, HT-29	16	TLK2, RPS3A, THEM6, RALGAPA2, GFPT1, MYH14, RALGAPB. RPS6, HIST1H2BJ, CD2AP, RPL11, CAPZB, RALGAPA1, CAPZA2, MYO1C, RPS15
Liver	12	FGA, FGG, RCN1, F5, FGB, TGM2, UGT2B4, HSPA1L, VDAC2, VTN, ALDH2, HP
Kidney	39	PRPF40A, HSPD1, TOP2B, ACIN1, DDX1, CRYAB, RNPS1, TNS1, ALDH6A1, SRSF9, ATP5B, A0A5E4, TUBB3, ASS1, HSP1A, RPL6, TUBB2A, RPL36, TRA2A, RPL3, ACAA2, PKM, TUBB2B, RPS24, HSP90AB1, SRSF3, RPL14, SLC34A2, PC, COL6A3, HSPA8, YH11, SRSF4, RPL8, TRA2B, MVP, RPL4, SRSF10, RPL15
Intestine	41	RAB10, ACTB, YBX1, ANXA2, CLCA1, FMOD, TUBB, CPT1A, COPA, VIL1, RPS18, GSTA1, ARF4, GAPDH, RPL7, RPL18A, ITLN1, UGT2B17, MYL12B, MEP1A, MYO1D, ACTC1, CALM1, RPL27, CPS1, RPL18, AHSA1, MEP1B, MYO1A, PDIA3, RPS23, RPL9, TUBB4B, TRIM21, CHMP4B, CLDN3, RPS8, TUBA1B, RPS15A, CLINT1, UGT2A3
HT-29	80	C10orf2, TPM3, SNRNP200, PKP3, CDK1, MAP4, ABCD3, PFKP, SLC25A10, CLMN, U2SURP, SMN1, SLC25A3, HSD17B10, HNRNPH2, RAB1B, COPG1, NDUFA10, PGAM5, PRPF8, COPB1, PHB2, IARS, DDX5, PRPF6, EHD4, BRAP, TCP1, RPS4X, GCN1L1, SPECC1L, SH3KBP1, PALD1, ATP2A2, RPS14, RPN1, CAPZA1, RPS7, EFTUD2, DDX23, CTNND1, ZNF609, HNRNPU, DARS, UBN1, PLEC, ABCE1, SLC25A6, CCT3, CABIN1, RPL22, LUZP1, EPRS, HIRA, KIF1B, UBN2, DHX40, DDX20, PYGB, MYH10, ATAD3A, SLC1A5, DHX15, RPS19, RPL27A, RBM17, RPS13, RBM14, RAN, MCCC2, PPIL4, AHNAK, NUP205, EIF4A1, RARS, SLC25A1, NSRP1, MCM7, DAPK3, ZNF638

*A unique UGT2B7 peptide (ADWLIR, 3 spectra) was also detected in intestine at a 95% peptide probability, 50% protein probability threshold
